# Protein Complex Detection via Weighted Ensemble Clustering Based on Bayesian Nonnegative Matrix Factorization

**DOI:** 10.1371/journal.pone.0062158

**Published:** 2013-05-02

**Authors:** Le Ou-Yang, Dao-Qing Dai, Xiao-Fei Zhang

**Affiliations:** Center for Computer Vision and Department of Mathematics, Sun Yat-Sen University, Guangzhou, China; University of South Florida College of Medicine, United States of America

## Abstract

Detecting protein complexes from protein-protein interaction (PPI) networks is a challenging task in computational biology. A vast number of computational methods have been proposed to undertake this task. However, each computational method is developed to capture one aspect of the network. The performance of different methods on the same network can differ substantially, even the same method may have different performance on networks with different topological characteristic. The clustering result of each computational method can be regarded as a feature that describes the PPI network from one aspect. It is therefore desirable to utilize these features to produce a more accurate and reliable clustering. In this paper, a novel Bayesian Nonnegative Matrix Factorization(NMF)-based weighted Ensemble Clustering algorithm (EC-BNMF) is proposed to detect protein complexes from PPI networks. We first apply different computational algorithms on a PPI network to generate some base clustering results. Then we integrate these base clustering results into an ensemble PPI network, in the form of weighted combination. Finally, we identify overlapping protein complexes from this network by employing Bayesian NMF model. When generating an ensemble PPI network, EC-BNMF can automatically optimize the values of weights such that the ensemble algorithm can deliver better results. Experimental results on four PPI networks of Saccharomyces cerevisiae well verify the effectiveness of EC-BNMF in detecting protein complexes. EC-BNMF provides an effective way to integrate different clustering results for more accurate and reliable complex detection. Furthermore, EC-BNMF has a high degree of flexibility in the choice of base clustering results. It can be coupled with existing clustering methods to identify protein complexes.

## Introduction

Protein-protein interactions (PPI) are fundamental to the biological processes within cells [1]. Most proteins form complexes to carry out biological tasks [2]. Protein complexes can help us to predict the functions of proteins [3], [4]. There is evidence that many disease mechanisms involve protein complexes [5]. Therefore, in the post-genomic era, predicting protein complexes is crucial. To address this problem, several biological experimental methods have been developed for detecting protein complexes. For instance, Tandem Affinity Purification (TAP) with mass spectrometry [6] can capture stable protein complexes, whereas Protein-fragment Complementation Assay (PCA) [7] can be used to study temporal and spatial dynamics of protein interactions. However, as mentioned in [2], [8], these methods have some inevitable limitations such as too much time consuming. Due to these experimental limitations, it is quite necessary to develop computational approaches which can be acted as useful complements to the experimental methods for detecting protein complexes.

Recently, high-throughput methods such as two-hybrid systems[9] and mass spectrometry [10] have been developed to detect a large amount of protein interactions, which enable the construction of PPI networks and make it possible for us to understand the cellular organization from the network level. A PPI network can be generally modeled as an undirected graph, where nodes represent proteins and edges represent pairwise interactions. Previous studies analyzed the graph topology of PPI networks and discovered that dense regions of the network may represent complexes [11]–[13]. These observations indicate the rationality of identifying protein complexes by detecting clusters from a PPI network.

In recent years, a vast number of computational approaches based on graph clustering have been applied to PPI networks for protein complexes identification. These graph clustering algorithms mainly depend on the structure topology analysis of PPI networks to identify protein complexes, which can be roughly divided into three categories: density-based approaches, graph partition-based approaches and hierarchical clustering algorithms. Several comprehensive reviews can be found in [2], [8], [14], [15]. The clustering result of a graph clustering algorithm is a set of clusters. In PPI networks, these clusters correspond to two types of modules: protein complexes and functional modules. A protein complex is a group of proteins that interact with each other at the same location and time. A functional module consists of proteins that participate in the same biological process or perform the same cellular function while binding each other at the same or different location and time [2], [15]. Here, we do not distinguish protein complexes from functional modules since we only use the PPI network as the underlying dataset for the mining task and the protein interaction data under consideration do not provided temporal and spatial information.

Unfortunately, due to the complex structure of the PPI network, the inner structure of protein complexes is still elusive. Given a PPI network, different protein complex identification algorithms may obeys different optimization criteria and yield diverse clustering results since each of them has been developed to capture one aspect of the network and neglect other network properties. Furthermore, the observed PPI networks obtained from high-throughput methods are quite noisy and therefore they may not represent the real situation. The performance of each protein complex identification algorithm heavily depends on network characteristics.

In fact, it is hard to find a protein complex identification algorithm that can generally work well for various networks with diverse properties [16]. Each algorithm has its own advantages and limitations: density-based approaches focus on detecting densely connected subgraphs in PPI networks. A typical example in this category is CFinder [17] which detects the *k*-clique percolation clusters as complexes. However, true complexes in the organism are not limited to densely connected substructures [1]. As pointed out by Qi *et al.* [1], complexes with sparsely connected substructures also exist in the PPI network. Therefore, traditional density-based approaches may ignore many biological meaningful complexes with low density. Additionally, due to the lack of global measurement, density-based algorithms can not produce satisfactory results. Graph partition-based approaches such as MCL [18] and RNSC [19] explore the best partition of a network. These algorithms are not able to discover overlapping complexes since they only support hard clustering. However, it is generally accepted that some proteins may perform different biological functions while interacting with different partners. Thus graph partition-based approaches cannot accurately capture the real structure of complexes in PPI networks. Hierarchical clustering algorithms [20], [21] can discover the hierarchical structure in a PPI network, which is important for understanding the global structure of functional organization. However, both bottom-up and top-down hierarchical approaches are sensitive to noisy data, whereas it is well known that the interaction data obtained from high-throughput methods may be quite noisy and contain a considerable fraction of false positives [22]. Furthermore, like graph partition-based approaches, hierarchical approaches cannot generate overlapping clusters [23] either.

In order to generate more reliable solutions, protein complexes identification algorithms should ideally exploit all features of the network and account for properties of the partitions, like overlaps and hierarchy. However, very few algorithms are capable of taking all these factors into consideration [24]. Note that the clustering result of each algorithm can be regard as a feature of the PPI network, which describes the network from one aspect. A natural question is whether we can utilize these features. Thus, we study a basic problem in this paper: provided that a PPI network is described by several clustering results computed from different computational methods, how to integrate these clustering results for accurate and reliable complex detection?

Ensemble clustering [25], [26] is a well known data analysis technique to address this problem. In machine learning literature, ensemble clustering has been proposed as an effective approach to strengthen the quality of simple clustering algorithms. There are reasons to believe that ensemble clustering may benefit from the integration of base clustering results. Hence, we would like to apply ensemble clustering to detect protein complexes in PPI networks. In this paper, the base clustering results are obtained from the application of different protein complex identification algorithms on the same PPI network. However, most existing ensemble clustering algorithms focus on naive combination frameworks. That is, they treat each base clustering result equally. But given a PPI network, some clustering results may be more reliable while others may be less reliable. Thus, different base clustering results should not be treated equally.

In light of the aforementioned challenges, to effectively utilize the information contained in different clustering results, we introduce a weighted ensemble approach which assigns a weight to each base clustering result. But there is not prior information to decide the values of these weights. Inspired by agglomerative fuzzy *k*-means clustering algorithm [27] and ensemble manifold regularization [28], we would like to automatically determine the values of these weights through an optimization process such that the ensemble clustering can produce better quality solution.

Clustering analysis by nonnegative matrix factorization (NMF) [29] has achieved remarkable progress in the past decade. Recently, it has been employed in cancer class discovery and gene expression analysis [30]. As a matrix decomposition techniques, NMF produces a low-dimensional approximation of a nonnegative matrix, in the form of nonnegative factors, which can be formulated as 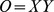. The nonnegativity of these factors allow them to be interpreted as a soft clustering of the data. As a clustering algorithm, how to estimate the optimal number of clusters (columns of *X* or rows of *Y*) is still a serious issue for NMF. Tan and Févotte [31] formulated a Bayesian approach to determine the effective number of columns of *X* (or rows of *Y*) via automatic relevance determination [32]. Recently, Psorakis *et al.* [33] applied this model on social networks for community detection. Compared with the previous algorithms, Bayesian NMF model has several advantages: first, each node is associated with a membership distribution over communities, which represent its propensity of belonging to each community. Therefore, it supports the overlap between communities. Second, it does not suffer from the resolution limit. Third, it is easy to implement and fast enough for large data sets. However, simple application of Bayesian NMF model on PPI networks may not obtain competitive results since many protein interactions detected by high-throughput methods may be false positives which will mislead the detection of complexes.

With these motivations, in this paper, we propose a novel Bayesian Nonnegative Matrix Factorization-based weighted Ensemble Clustering (EC-BNMF), for the purpose of identifying protein complexes. EC-BNMF can integrate multiple clustering results (features) of a PPI network and produce a more accurate and informative clustering. In addition, EC-BNMF allows proteins to be shared among complexes, which is much closer to the reality. By applying EC-BNMF on four yeast PPI networks, we show that EC-BNMF has competitive performance with the state-of-the-art algorithms in detecting protein complexes. Furthermore, the experimental results well verify the effectiveness of EC-BNMF in detecting multi-functional proteins.

### Related Works

In recent years, several approaches based on ensemble clustering have been applied to PPI networks for the purpose of detecting protein complexes [34], [35]. To weight edges of the PPI network and measure the reliability of the corresponding interactions, Asur *et al.* [34] first introduced two similarity metrics-clustering coefficient-based metric and betweenness-based metric. After improving the quality of the data, they used three conventional graph partition-based algorithms-repeated bisections, direct *k*-way partitioning and multilevel *k* -way partitioning to generate six base clustering results. These base clustering results all consist of *k* clusters, here *k* was the predefined number of clusters of each base clustering. Then they described two different techniques-pruning and weighting to eliminate noisy clusters. Finally, they developed a consensus method based on principal component analysis to solve the clustering problem. In order to discover multi-functional proteins, they also designed an adaptation to allow for soft clustering. However, the base clustering algorithms are all partition-based methods. Thus they may not be able to fully capture the structure of the network, and their performance may heavily depends on the quality of the two similarity metrics. What is more, the base clustering algorithms and the consensus algorithm all need to predefine the number of clusters, but the true number of complexes is always unknown.

Another ensemble framework for detecting protein complexes was proposed by Greene *et al.* [35]. With different number of dimensions, they first generated a collection of non-negative matrix factorizations. Then they proposed a hierarchical meta-clustering algorithm to aggregate these factorizations and produce a disjoint hierarchy of meta-clusters. Finally, they transformed these results into a soft hierarchical clustering of the original dataset. Most recently, Lancichinetti and Fortunato [36] presented a systematic study of consensus clustering in complex network. They demonstrated that consensus clustering can be used to cope with the stochastic fluctuations in the results of clustering techniques. Given a network 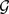 and a clustering algorithm *S*, they first applied *S* on 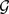 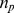 times and obtained 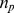 partitions. Then they computed a consensus matrix *D* which was based on the cooccurrence of nodes in clusters of the base partitions. After filtering out small entries in *D*, they applied *S* on *D* 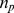 times and produced 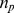 partitions again, which could generated a new consensus matrix. The procedure is iterated until a unique partition is reached, which cannot be altered by further iterations. Both these two algorithms focus on generating more accurate and stable results out of a set of partitions delivered by a specific method. Greene *et al.* developed an algorithm to identify protein complexes from several clustering results, but they did not do selection among base clustering results. Whereas Lancichinetti and Fortunato extracted reliable information from base clustering results and used the original algorithm to detect communities.

## Methods

Given a PPI network with *N* proteins, we use an undirected simple graph 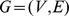 with a set of nodes *V* and a set of edges *E* to model it, where nodes represent proteins and edges represent pairwise interactions. The graph can be represented by an adjacency matrix *A*, where 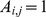 if there is an edge between protein *i* and *j*, and 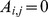 otherwise. In this way, the problem of detecting protein complexes is cast into clustering the nodes into groups.

The task of ensemble clustering is to obtain a comprehensive consensus clustering by integrating 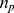 diverse and independent clustering results (here we call them base clustering results): 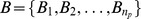. Each base clustering result 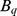 is generated by a computational algorithm (here we call them base clustering algorithms). As some of the base clustering results do not cover all proteins in the PPI network (i.e., MCODE), we set each of the unclustered proteins to be a singleton cluster. Therefore, each base clustering result contains all of the proteins in the PPI network. Given a PPI network, there are several ways to obtain a collection of clustering results. They can be generated by a given approach with different initializations, or from different approaches.

**Figure 1:**
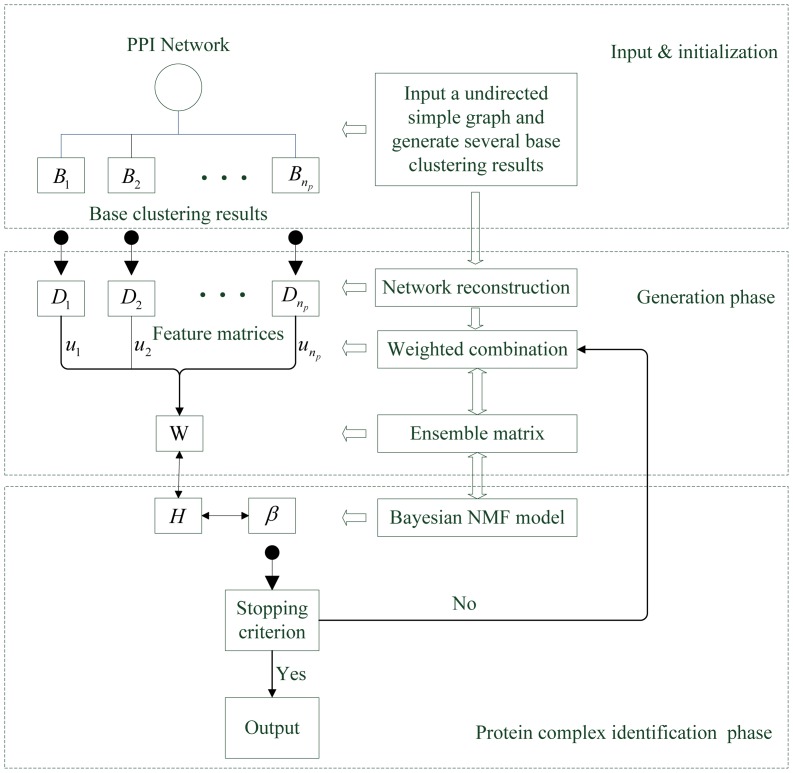
Schematic overview of EC-BNMF. EC-BNMF consists of two phases: a generation phase which integrates several base clustering results into an ensemble PPI network and a complex detection phase which accomplishes the detection of protein complexes.

In this section, we propose a novel Bayesian Nonnegative Matrix Factorization-based weighted Ensemble Clustering (EC-BNMF) to perform efficient protein complexes detection. EC-BNMF consists of two phases: a generation phase which extracts useful information from several base clustering results and generates an ensemble PPI network, and a complex detection phase in which a Bayesian NMF-based ensemble clustering is employed to detect protein complexes from the ensemble PPI network. The flow-chart of the algorithm is shown in Figure 1.

### Constructing an ensemble PPI network

Given an original PPI network and a collection of base clustering results, the goal of this phase is to extract useful information from these data. Here, each base clustering result is regarded as a “feature” of the original PPI network, which provides a description of this network. In order to analyze these “features” from network perspective, we construct a feature network through each base clustering result. In each feature network, two proteins are connected if they have cooccurred in the same cluster at least once. Therefore, if we buy the popular definition of protein complexes as subgraphs with a high internal edge density and a low external edge density, it is easy to identify protein complexes from the feature network since it just consists of a set of fully connected subgraphs. Furthermore, since the original PPI network contains important information, we also treat the original PPI network as a feature network. In this way, we obtain 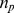 feature networks that describe the original PPI network from different aspect.

We use a feature matrix 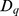 to represent the adjacency matrix of the *q*-th feature network. According to the definition of the feature network, each entry of 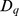 denotes whether the corresponding pair of proteins has been clustered together. Thus 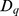 is a block-diagonal matrix after some permutations (except the adjacency matrix of the original PPI network). As mentioned above, the goal is to extract useful information from these feature networks and generate an ensemble PPI network that is rich in information. Thus the task is turned into the problem of combining these feature matrices into an ensemble matrix *W* which corresponds to an ensemble PPI network. To effectively utilize the information provided by these feature networks, we propose a novel weighted combination framework to generate the ensemble PPI network. We hope that the ensemble PPI network can approximate the intrinsic of the original PPI network, thus we propose an alternative approach by assuming that the ensemble PPI network is a weighted combination of these feature networks. The above assumption is equivalent to the following constrain:
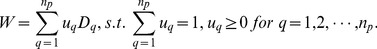
(1)


Here *W* is an ensemble matrix corresponding to the ensemble PPI network, and 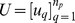 is a vector of weights. Therefore, the problem of generating an ensemble PPI network is turned into the problem of learning the optimal linear combination of the feature matrices.

In order to avoid the parameter *U* overfitting to one feature network (one of the feature networks is weighted at 1 and all other feature networks are weighted at 0), we introduce a regularization term 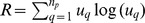 which represents the sum of the negative entropy of the weight for each feature matrix. It can penalize solutions with maximal weight on a single feature network. We will later show how to automatically estimate the optimal weights.

### Detecting protein complexes from the ensemble PPI network via Bayesian NMF-based clustering algorithm

It is worthy to stress that 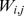 represents the evidence provided by base clustering results that protein *i* and *j* belong to same complexes. Therefore, in the ensemble PPI network, the stronger the interaction between two proteins, the more likely they perform the same biological functions. In other words, if two proteins have strong interaction, they have high propensities on the same complexes. Based on the characteristics of the ensemble PPI network, we develop a Bayesian NMF-based clustering algorithm to detect protein complexes from this network, which can utilize the group information provided by the edges. In this section, we outline the main idea of this algorithm.

Assuming we have obtained the ensemble matrix *W* through the generation phase, then the task is to detect protein complexes from the ensemble PPI network to which *W* corresponds. In other words, given a protein, we attempt to exploit the groups it belongs to. Since such group memberships are always unknown, we can only infer them from the observed network. Here, each entry 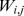 of *W* denotes the nonnegative count of interactions between proteins *i* and *j*. Suppose there are *K* complexes in the PPI network. For each protein *i*, similar to [33], we introduce a parameter 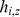 to indicate the strength of protein *i*'s membership of complex 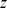. A higher value of 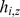 means protein *i* is more likely in complex 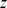. The important point is that protein *i* may have high value of 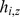 on more than one complexes, thus our method allows proteins to belong to multiple complexes. Furthermore, not all of the complexes need to have proteins associated with them, hence *K* just represents the upper bound on the number of complexes.

Let 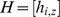 be the protein-complex propensity matrix. According to the definition of 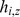, the value of 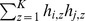 represents the possibility of protein *i* and *j* belong to the same complexes. As we have mentioned above, the value of 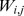 also represents the evidence that protein *i* and *j* should be clustered together. Therefore, the pair-wise interactions described in 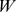 are affected by these unobserved nonnegative parameters which can be described as 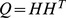, where 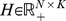. Each element 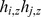 indicates the contribution of complex 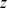 to 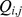. Similar to [31], [33], [37], we assume that the likelihood of a single element 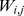 of the matrix 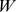 is given by 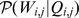, where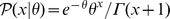 is the Poisson probability density function with rate 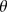.

In practice, given a network, the number of complexes is initially unknown. To ameliorate this problem, as presented in [31], [33], [37], we place automatic relevance determination [32] priors 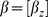 on the columns of *H*. The effect of these priors is to pick up relevant columns of *H* that could best account for the observed interactions.

Following the generation model described above, we write down the probability that an ensemble PPI network is generated:
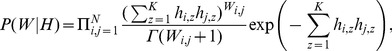
(2)where *W* is the ensemble matrix. Following the choice of [31], [37], we assign independent Half-Normal priors on each column of H with zero mean and variance 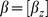:
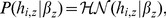
(3)where for 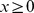, 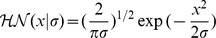, and for 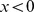, 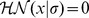. From Equation (3) we find that the elements of the 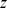-th column of *H* are associated with a variance-like parameter 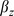 (also known as the relevance weight), which controls the relevance of the corresponding complex in accounting for the observed interactions. When the value of 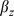 is small, all the elements of the 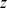-th column of *H* are close to zero, which means this column is irrelevant and can be removed from the factorization. Through this filter, we obtain a more parsimonious model which indicates the optimal number of clusters.

Similar to the model used in our previous works [38], [39], the generative model introduced above will be sensitive to the choice of 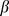. To alleviate this problem, under the assumption that each 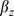 are independent, each relevance weight 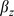 is given an inverse-Gamma priors which is conjugate to the Half-Normal distribution. Therefore the joint distribution of 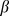 will be:
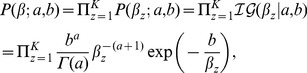
(4)where *a* and *b* are the (nonnegative) shape and scale hyperparameters respectively. We set *a* and *b* to be constant for all 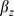. In this way, the model may not very sensitive to the choice of *a* and *b*. Take all these factors into consideration, we adopt a Bayesian network model to describe the generation process of an ensemble PPI network, and the resulting product is of the form:
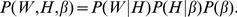
(5)


**Figure 2:**
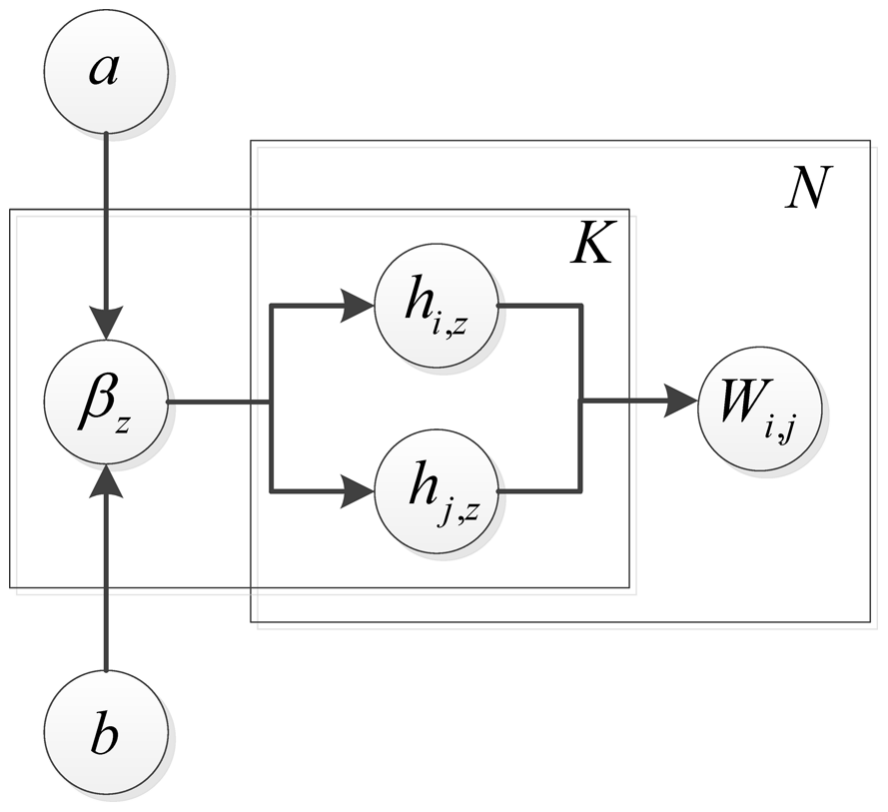
Graphical representation of the dependence between parameters. A graphical model that describes the generation process of an ensemble PPI network with weighted adjacency matrix *W* in terms of the latent structure *H*, the components of which are generated using half-normal distribution with zero mean and relevance weights 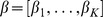. The rectangles are used to group random variables that repeat. The number of repetitions is shown on the top right corner.

For an ensemble PPI network, we estimate the values of *H* and 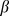 by maximum the joint probability of Equation (5). By taking Equations (2),(3) and (4) into Equation (5), and taking the negative logarithm and dropping constants, we obtain the objective function of Bayesian NMF-based clustering algorithm:
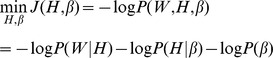


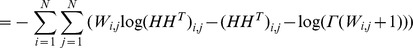


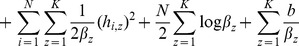
(6)

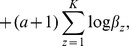


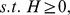
where 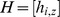 is the protein-complex propensity matrix. A graphical model to describe the dependence between all these parameters are illustrated in Figure 2. Since 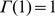 and 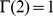, and the value of 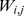 is between 0 and 1, we assume 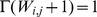 for simplicity. Therefore, the term 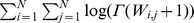 will disappear.

### Protein complex detection via Bayesian NMF-based weighted Ensemble Clustering

Integrating the above two phases, we obtain a novel Bayesian NMF-based weighted Ensemble Clustering algorithm. The constructed ensemble PPI network is a weighted undirected network and each element 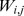 of its adjacency matrix *W* represents the probability of protein *i* and *j* belonging to the same complex. Bayesian NMF model assumes that the joint membership of two proteins in the same complex raises the probability of a link existing between them. Therefore, it can effectively identify protein complexes from the ensemble PPI network. Next, we first introduce the objective function of Bayesian NMF-based weighted Ensemble Clustering algorithm. Then we discuss how to optimize this model and estimate the value of the model parameters. Finally, we use this model to detect protein complexes from PPI networks through the estimators of these model parameters.

#### Objective function of Bayesian NMF-based weighted Ensemble Clustering

Adding the introduced regularizer *R* to the objective function (6), and substituting *W* with Equation (1), then we present a novel weighted ensemble clustering algorithm-Bayesian Nonnegative Matrix Factorization-based weighted Ensemble Clustering (EC-BNMF):
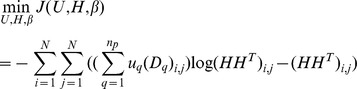


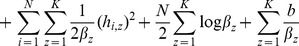
(7)

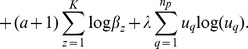


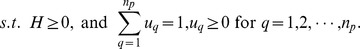



Here coefficient 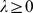 is the tradeoff parameter which controls the balance between objective function (6) and regularizer *R*.

#### Solution to Bayesian NMF-based weighted Ensemble Clustering

Minimization of 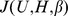 in (7) with the constraints form a constrained nonlinear optimization problems. To optimize 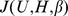, similar to [27], we alternately update *H*, 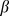 and *U*. In this procedure, we first fix the values of *U*, and optimize the value of *H* and 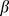. Then we fix *H* and 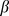, and optimize the value of *U*. We repeat this alternate updating procedure until the solution converges. In the following we describe the details.

Given *U*, (7) degenerates to (6), thus we minimize 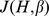 with respect to *H* and 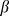. Similar to [31], [33], we adopt the multiplicative update rule [29], [40] to estimate *H* and 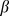, which is widely accepted as a useful algorithm in solving nonnegative matrix factorization problem.

By the multiplicative update rule, we obtain the following two updating rules for 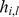 and 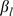:
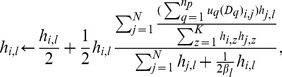
(8)and
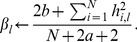
(9)


After an update of the values of *H* and 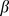, we fix *H* and 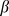, and turn to the update of *U* with respect to (7). By solving the constrained optimization problem, we obtain the following updating rule for 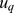:
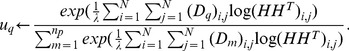
(10)


The updating rules (8) can maintain the nonnegativity of the parameters to be inferred. The elements of *H* will always be nonnegative during the iteration if we initialize *H* with nonnegative values. For the detailed inference of the three updating rules, please refer to Text S1.

#### From protein-complex propensity matrix to protein complexes

Observing at the updating rule (9), it is obvious that each 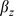 is bounded from below by 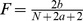 during each iteration, and it will attains this bound when the 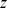-th column of 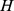 is a zero vector, which means the 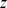-th complex is pruned out of the model. After convergence, we set 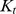 to be the number of complexes which satisfy the following condition:
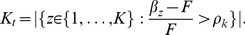
(11)


Here, 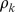 is a threshold that need to be predefined. Therefore, if 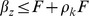, the 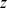-th column of *H* is regarded as irrelevant complex, and could be filtered out. As mentioned above, each column of *H* contains *N* nonnegative real values presenting each protein's degree of participation into the corresponding complex. After computing 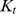 and filtering out irrelevant columns of *H*, similar to [35], [38], [39], we obtain protein complexes from *H* by taking a threshold 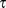 and assigning a protein to a complex if its membership weight for that complex exceeds 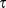. In this way, we obtain the resultant protein-complex membership matrix 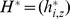, where 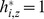 if 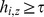 and 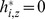 if 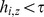. Here, 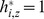 means protein *i* is assigned to detected complex 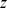. Similar to [41], we only consider the identified complexes that have at least three members since the complexes with two proteins have been presented in the protein interaction data. After completing these steps, we obtain the optimal number of complexes 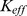 which represents the number of columns of H^*^ that contain at least three elements of 1.

#### Final algorithm

**Figure 3:**
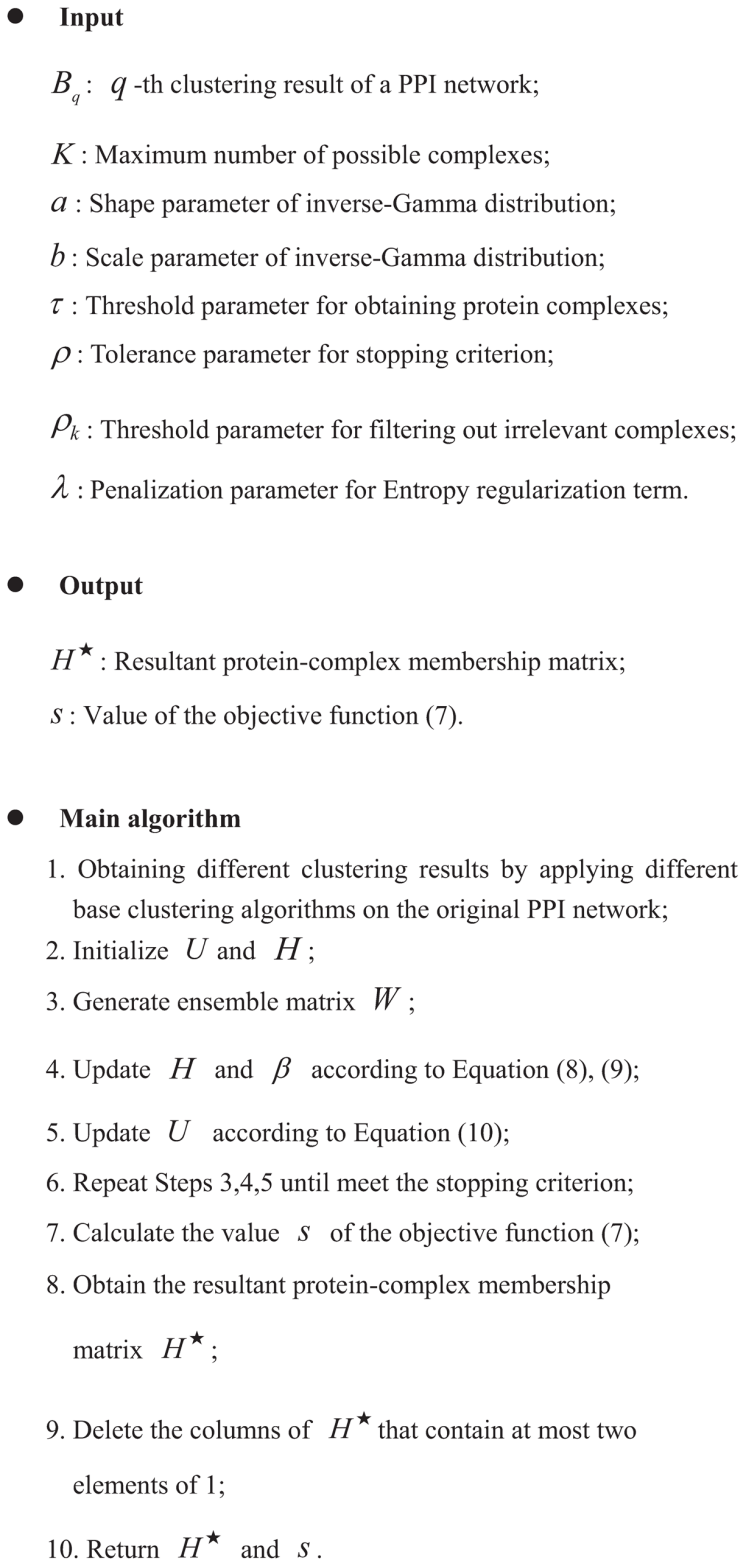
Summary of EC-BNMF for detecting protein complexes.

We summarize the overall algorithm in Figure 3. In this paper, we iteratively update *H*, 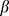 and *U* according to the updating rules (8), (9) and (10) until they satisfy a stopping criterion. Let 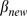 and 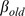 be the vector of relevance weights at the current and previous iterations respectively. The algorithm is stopped whenever 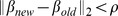, where 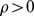 is a user defined tolerance parameter. For simplicity, we set the value of this tolerance parameter to be the same as the threshold 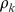. Furthermore, we limit the calculation procedure to a maximum of 150 iterations for practical purposes. That is, we stop iterating when 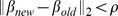 or the number of iterations reach 150. Here, the typical value of 1E-6 is selected as the value of the tolerance parameter 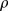 and the threshold 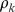. In order to avoid a local minimum, we repeat the algorithm 50 times with random initial conditions and choose the result that outputs the lowest value of objective function (7).

Here, we also consider two special cases of our model. First, if we fix the value of each weight 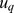 to be 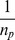, the ensemble matrix is 
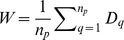
, thus the weighted combination framework degenerates to the naive combination framework. In this case, each feature network is treated equally. Second, if the weight of the original PPI network is set to 1, the ensemble matrix *W* becomes *A* which is the adjacency matrix of the original PPI network. In this case, our model is equivalent to applying Bayesian NMF model on the original PPI network.

## Results

In this section, we evaluate the effectiveness of EC-BNMF in detecting protein complexes. Before presenting the results of our comparative experiments, we first describe the PPI networks and validation metrics that are used. Then we discuss the effect of parameters and the benefits of weighted ensemble clustering. Next, we investigate the performance improvement brought by integrating diverse clustering results. Finally, we compare EC-BNMF with other ensemble clustering algorithms and evaluate the overlapping protein complexes detected by EC-BNMF.

### PPI networks

EC-BNMF is tested using four PPI networks from S. cerevisiae. The yeast S. cerevisiae is a highly effective model organism that presents an ideal opportunity to test the performance of a newly proposed algorithm since a great deal of protein complexes of it is known. In this paper, we concentrate our analysis on the following four different high-throughput derived PPI networks: a high-reliable database published by Collins *et al.* [42], two experimental yeast PPI networks published by Gavin *et al.* [43] and Krogan *et al.* [44] respectively, and the entire set of physical interactions in yeast from BioGRID [45], [46]. Here we use Collins, Gavin, Krogan and BioGRID to represent these four networks. In this paper, for simplicity, we just extract the largest connected components from all the four networks. The corresponding features of the four networks are listed in Table 1. As can be seen from this table, these four networks have different topological properties, we use them as model datasets to test the comprehensive performance of EC-BNMF.

**Table 1:** Topological characteristics of the used PPI networks.

	Collins	Gavin	Krogan	BioGRID
Number of proteins	1004	1359	2559	5850
Number of interactions	8319	6451	7031	68312
cc	0.6478	0.4196	0.1947	0.2622
avNeighbors	16.57	9.49	5.50	23.35
density	0.0165	0.0070	0.0021	0.0040

Here cc denotes the average clustering coefficient of network, avNeighbors denotes the average number of neighbors of each protein.

### Gold standard protein complexes

To measure the accuracy of the detected complexes, we choose two widely used benchmark complex reference sets as gold standards. One of them is downloaded from the MIPS database [47], the other one is derived from the Gene Ontology annotations of the Saccharomyces Genome Database [48], [49]. Following Brohée and Van Helden's study [14], we use the 220 filtered yeast protein complexes from MIPS database as our first reference set, and we call them MIPS complexes here. In addition, since the complexes in MIPS database do not cover all the proteins in the considered network, we also use another independent reference set, and we call them SGD complexes here. SGD complexes are generated from SGD database [48] following the procedure described by Nepusz *et al.* [41].

The MIPS complexes are download from http://rsat.bigre.ulb.ac.be/rsat/data/publisheddata/brohee_2-006_clustering_evaluation/index_tables.html/. The SGD annotations and GO structure are download from Gene Ontology database [50] http://www.geneontology.org/on 24 April 2012. In order to prevent the membership of the same protein inconsistencies, we test these two reference set separately. For both reference sets, to avoid selection bias, we filter out the proteins that are not contained in the network at hand. Furthermore, only complexes with at least 3 and no more than 100 members are considered. In Table 2 we summarize the statistics of these reference sets with respect to each PPI network.

**Table 2:** Gold standard protein complexes.

Network	Reference database	# complexes	# proteins
Total	MIPS	220	1095
	SGD	324	1340
Collins	MIPS	64	437
	SGD	81	426
Gavin	MIPS	94	537
	SGD	118	542
Krogan	MIPS	119	601
	SGD	168	790
BioGRID	MIPS	157	1010
	SGD	242	1217

Here “Total” denotes the statistics of each reference database which are not mapped into a special PPI network and filtered by size.

### Evaluation criteria

We evaluate the performance of a protein complex identification algorithm by judging how well the predicted complexes correspond to the known complexes. In this study, three independent quantity measures are used to assess the similarity between a set of predicted complexes and a set of reference complexes. The first one is the *f*-measure which is defined as the harmonic mean of Precision and Recall [1]. The other two are the Jaccard and PR metrics which are proposed by Song and Singh [16]. Among these three measures, *f*-measure is used to assess the similarity between predicted complexes and reference complexes at complex level (Recall measures what fraction of the reference sets are matched by the predicted complexes, and Precision measures what fraction of the predicted complexes are matched by the reference complexes). Whereas Jaccard and PR metrics can measure how well the predicted complexes correspond to reference complexes at complex-protein pair level, which take into account the number of proteins in each complex. The value of each measure vary between 0 and 1, and the higher value means better overlaps. For more details about these three scoring measures, please refer to Text S2. These evaluation metrics can provide us some sense of how well the protein complex identification algorithm can be used to detect protein complexes from PPI networks.

### Choice of parameters

There are five parameters *K*, 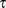, *a*, *b* and 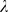 that need to be predefined in our algorithm. *K* is the maximum number of complexes. Note that EC-BNMF can filter out irrelevance complexes, so the value of *K* can be taken sufficiently large. Here we empirically set 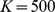 for Collins, Gavin and Krogan, and 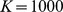 for BioGRID. 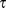 is the threshold used to obtain protein complexes from the protein-complex propensity matrix, and we find experimentally that 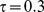 always leads to reasonable results on the four networks. Observing that the shape hyperparameter *a* affects the optimization of the objective function (7) only through the updating rule (9), thus the influence of *a* is moderated by the number of nodes *N*. Therefore, we choose *a* to be small compared to *N*. Experimental results also confirm that smaller value of *a* leads to better results. In this paper, we fix 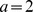 and vary the value of *b* to find the best result for each network. Another key parameter is 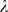 which control the effect of regularization term 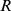. The parameter 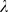 controls the relative differences between feature networks. Setting 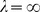 forces all feature networks to be given equal weight, whereas setting 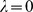 discards the regularization term. Through updating rule (10) we can find that the effect of 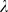 depends on the value of 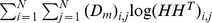. In order to facilitate the selection of 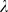, we set the value of 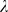 to be in proportion to 
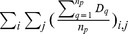
. That is, we set 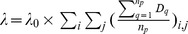. To find out the suitable value of 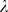, we just need to vary the value of 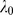 and evaluate the corresponding performance. Finally, the key parameters that affect the performance of EC-BNMF are *b* and 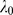.

In order to fully understand how these two parameters affects the performance, we investigate how the performance changes as the values of these parameters change. To this end, we vary the values of *b* and 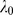 for each PPI network, and compare the corresponding experiment results in terms of Jaccard, PR and *f*-measure with respect to two reference sets. For each PPI network, we try different combination values of 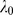 (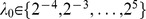) and *b* (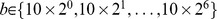).

**Figure 4:**
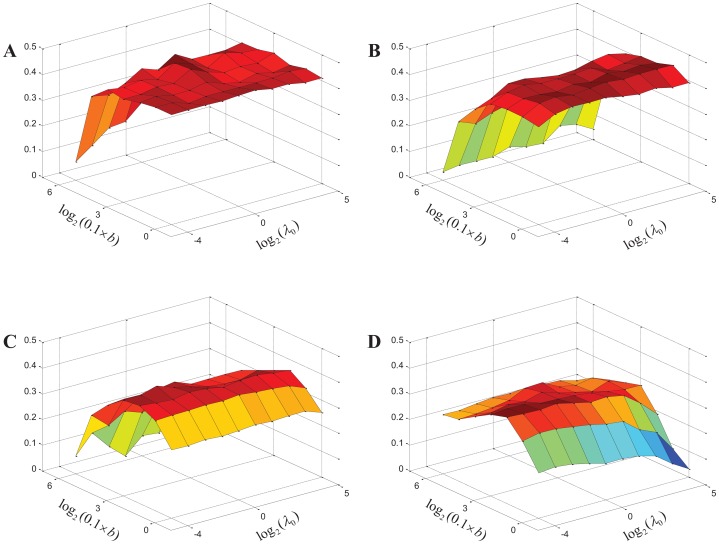
Effect of parameters. Performance of EC-BNMF on protein complex detection with respect to different values of *b* and 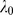 measured in terms of the harmonic mean score. The 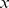-axis denotes the value of 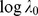, the 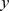-axis denotes the value of 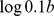, and the 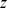-axis denotes the value of the harmonic mean of the three measure scores of both MIPS and SGD complexes. (A) Collins network. (B) Gavin network. (C) Krogan network. (D) BioGRID network.

For each network, the harmonic mean of six scores (Jaccrad, PR and *f* -measure with respect to MIPS and SGD complexes) is used to measure the performance of EC-BNMF. Figure 4 shows the corresponding results with respect to various values of 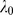 and *b* on the four PPI networks. As shown in Figure 4, for a fixed value of 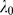, as the value of *b* increases, the harmonic mean scores increase initially and decrease after reaching the maximum, and this is true for all the four PPI networks. On the other hand, for a fixed value of *b*, as the value of 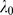 increases, the harmonic mean scores increase initially and decrease after reaching the maximum, but the change is not very obvious. This phenomenon is partly owing to the choice of prior information 
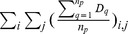
. With this prior information, the model is not very sensitive to small changes in 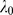. In fact, if 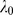 is large enough, the weight assigned to each feature network is nearly equal. Thus the performance will not change a lot with the increase of 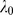. In our model, 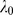 is used to adjust the weights, and it only affects the quality of the ensemble PPI network. Unless a considerable fraction of base clustering results are poor, a small change of 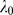 can not lead to big change of the performance. From Figure 4, we can find that in terms of parameters, EC-BNMF is relatively stable. In fact, the performance of EC-BNMF is very sensitive to the choice of 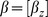. To reduce the sensitivity, we assign independent inverse-Gamma priors on each 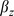 such that EC-BNMF can automatically estimate the optimal value of 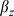. Through the updating rule (9), EC-BNMF can adaptively adjust the value of 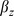. Thus, EC-BNMF is not very sensitive to the choice of *b*. Nevertheless, both *b* and 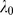 contribute to improving the performance of EC-BNMF.

We can find from Figure 4 that the optimal result are obtained when 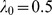 and 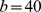 for Collins network, 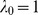 and 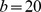 for Gavin network, 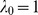 and 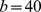 for Krogan network, and 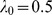 and 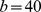 for BioGrid network. In the following, unless otherwise stated, the complexes detected by EC-BNMF are obtained with these optimal values of parameters for the four PPI networks.

### Performance evaluation

In this section, we systematically evaluate the proposed model on the protein complex detection task. EC-BNMF strives to combine several partitions of a network into a more desirable clustering result. These partitions can be obtained from a single clustering algorithm with different initializations or from the application of different clustering algorithms on a network. In this paper, we focus on the combination of the results of different algorithms since different algorithms may discover different patterns in a given network and increase the information available for ensemble clustering. Therefore, we choose ten state-of-the-art algorithms as base clustering algorithms: CFinder [17], CMC [51], ClusterONE [41], COPRA [52], DPClus [53], MCL [18], MCODE [12], MINE [54], RNSC [19], and SPICi [55]. A brief description of these algorithms and the setting of parameters are discussed in Text S3. We also list the websites where we download the corresponding softwares in Text S3 Table 1.

#### Weighted combination versus naive combination

In this section, we investigate the benefits of performing weighted combination when constructing the ensemble PPI network. As a baseline for comparison, we test the performance of naive combination which is a special case of EC-BNMF, where 
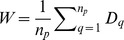
. Here, we call it naive ensemble clustering (NEC).

**Figure 5:**
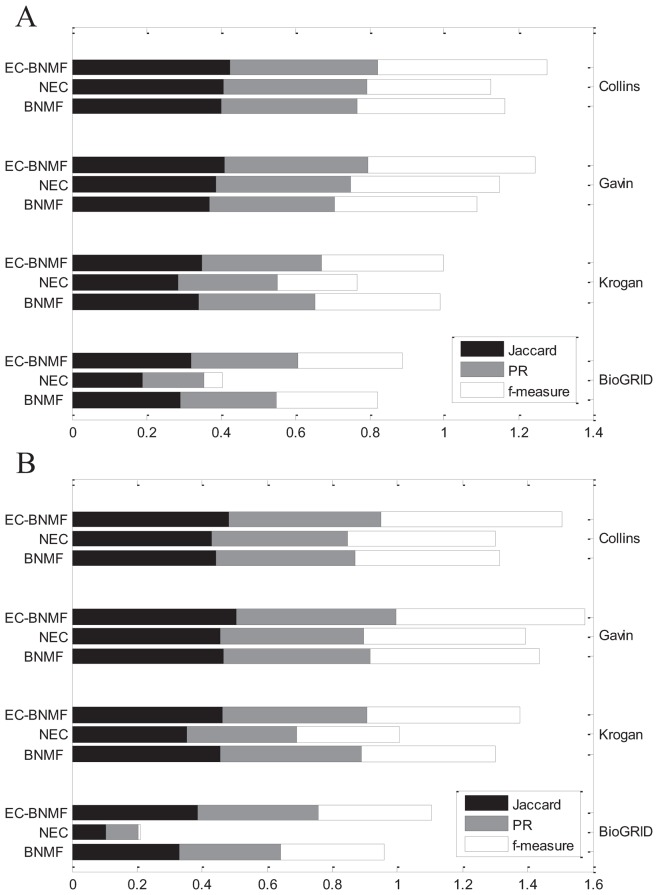
Comparative performance of applying Bayesian NMF model on weighted (naive) ensemble networks and original PPI networks. Comparison of the performance of EC-BNMF, naive ensemble clustering (NEC) and Bayesian NMF model (BNMF) on four PPI networks with respect to (A) MIPS gold standard and (B) SGD gold standard.

We apply EC-BNMF and NEC on four PPI networks, and compare their performance. Through updating rules (8) and (9), we can find that the performance of NEC depends on the choice of parameter *b*. For each PPI network, the results of NEC are obtained over the best tuned parameters. Figure 5 shows the comparative performance of EC-BNMF and NEC on four PPI networks in terms of the three measures (Jaccard, PR and *f*-measure) according to MIPS and SGD complexes. From Figure 5, we can see that EC-BNMF leads to better performance on all the four PPI networks.

In real cases, the performance of each clustering algorithm is based on the topological characteristics of the network under consideration. Given a network and a collection of clustering results, some clustering results may perform well in recapitulating protein complexes while others may not. Therefore, constructing an ensemble PPI network by simply averaging is inadequate since the information provided by poor clustering results may be unreliable, and may affect the performance of ensemble clustering.

#### Ensemble PPI network versus original PPI network

To demonstrate the benefits of using the ensemble PPI network, we consider the individual performance of applying Bayesian NMF model (BNMF) on the original PPI network. That is, we assume 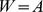 which is the adjacency matrix of the original PPI network. It is noteworthy to mention that Bayesian NMF model is also a popular clustering algorithm. Thus, it is of great interest to test the performance of Bayesian NMF model on original PPI networks. Next, we apply BNMF and EC-BNMF on four PPI networks, and compare their performance. According to updating rules (8) and (9), the performance of BNMF is based on the choice of *b*. For each PPI network, to be fair, the results of BNMF are obtained over the best tuned parameters. The comparison of these two algorithms are displayed in Figure 5.

The results shown in Figure 5 well verify the benefits of using ensemble PPI networks. It can be observed that EC-BNMF leads to better performance than the individually inferred by applying BNMF on the original PPI network. These results once again show that the performance of a complex identification algorithm is based on its optimization criteria and the characteristic of the network under consideration. BNMF is based on the assumption that if there is an edge between two proteins, they may belong to the same complex. However, the data obtained from high-throughput methods is believed to be quite noisy, and many interactions may be false positives. Therefore, if the network does not satisfy this assumption, the complexes detected by BNMF may be less reliable. In EC-BNMF, we integrate the results of different clustering algorithms, and generate an ensemble PPI network. Each edge in the ensemble PPI network represents the evidence (provided by base clustering results) that the corresponding proteins belong to same complexes. Thus Bayesian NMF model can find out more reliable complexes.

Furthermore, we can see from Figure 5 that BNMF has better performance than NEC on Krogan and BioGRID. In NEC, all base clustering results are treated equally, thus the information provided by unreliable clustering results may mislead the detection of complexes.

#### Quantitative comparison with base clustering algorithms

To further evaluate the competitiveness of EC-BNMF in detecting protein complexes, we compare its results with the ones from base clustering algorithms since these algorithms are also the most popular methods. For all the clustering results, we only consider clusters that have at least three elements. Table 3 presents the comparative performance of different clustering algorithms on four PPI networks. The details of these algorithms are presented in Text S3. The results are obtained over the best tuned parameters for each algorithm. Remarkably, for BioGRID, CFinder can not give a clustering result in 48 hours, so it does not take part in the generation phase when considering BioGRID dataset, and the corresponding result will not be listed in this table.

**Table 3:** Performance comparison of EC-BNMF and base clustering algorithms on detecting protein complexes.

Network	Algorithm	MIPS			SGD			weight(*u_n_*)
		Jaccard	PR	f-measure	Jaccard	PR	f-measure	
Collins	EC-BNMF	**0.4244**	**0.3984**	**0.4530**	**0.4811**	**0.4696**	**0.5547**	
								Original 0.1264
	CFinder	0.4038	0.3841	0.4224	0.4122	0.4056	0.4938	0.0313
	ClusterONE	0.3900	0.3640	0.4046	0.4079	0.3975	0.4965	0.0941
	COPRA	0.3570	0.3465	0.3868	0.3624	0.3543	0.5249	6.437e-6
	CMC	0.3871	0.3533	0.4068	0.4268	0.4139	0.5195	0.0802
	DPClus	0.3952	0.3641	0.3968	0.4421	0.4281	0.5168	0.1370
	MCL	0.3881	0.3685	0.3504	0.3871	0.3752	0.4928	0.0568
	MCODE	0.4189	0.3887	0.4287	0.4601	0.4472	0.5526	**0.1450**
	MINE	0.3942	0.3721	0.4033	0.4173	0.4066	0.5504	0.0512
	RNSC	0.4170	0.3882	0.4260	0.4519	0.4419	0.5529	0.1383
	SPICi	0.3953	0.3637	0.4490	0.4392	0.4246	0.5537	0.1397
Gavin	EC-BNMF	**0.4104**	**0.3851**	**0.4504**	**0.5048**	**0.4912**	**0.5780**	
								Original 0.0872
	CFinder	0.3446	0.3142	0.3505	0.4034	0.3906	0.4445	0.0616
	ClusterONE	0.3335	0.3050	0.3536	0.4537	0.4370	0.4824	0.1130
	COPRA	0.2636	0.2500	0.2844	0.2966	0.2883	0.3987	0
	CMC	0.3821	0.3060	0.2310	0.3683	0.3568	0.3326	0.0625
	DPClus	0.3594	0.3283	0.3824	0.4652	0.4465	0.5259	0.1315
	MCL	0.3506	0.3247	0.3333	0.4312	0.4127	0.4415	0.1066
	MCODE	0.3051	0.2659	0.3338	0.3682	0.3380	0.4489	0.1189
	MINE	0.3098	0.2724	0.3097	0.3610	0.3389	0.4288	0.0413
	RNSC	0.3586	0.3330	0.3286	0.4476	0.4331	0.4620	0.1324
	SPICi	0.3624	0.3318	0.3872	0.4769	0.4553	0.5483	**0.1449**
Krogan	EC-BNMF	**0.3491**	**0.3230**	**0.3258**	**0.4624**	**0.4442**	**0.4691**	
								Original 0.0943
	CFinder	0.2865	0.2639	0.2223	0.3828	0.3684	0.3331	0.1039
	ClusterONE	0.2780	0.2503	0.2813	0.4099	0.3896	0.4203	0.1029
	COPRA	0.1415	0.1281	0.1725	0.1826	0.1696	0.3183	0
	CMC	0.3322	0.3044	0.2754	0.4211	0.4033	0.3885	0.1040
	DPClus	0.3274	0.3001	0.3144	0.4542	0.4343	0.4671	**0.1051**
	MCL	0.2204	0.1917	0.1548	0.2945	0.2682	0.2802	0.0832
	MCODE	0.2682	0.2359	0.2262	0.3220	0.2937	0.3176	0.0988
	MINE	0.2946	0.2621	0.2664	0.3617	0.3373	0.3938	0.1020
	RNSC	0.2950	0.2705	0.2425	0.3934	0.3757	0.3894	0.1030
	SPICi	0.3203	0.2968	0.2784	0.4289	0.4125	0.4490	0.1047
BioGRID	EC-BNMF	**0.3206**	**0.2866**	**0.2823**	**0.3871**	**0.3688**	**0.3474**	
								Original 0.1110
	ClusterONE	0.2266	0.1889	0.1453	0.2974	0.2731	0.2898	0.1344
	COPRA	0.0194	0.0184	0.0245	0.0032	0.0032	0	0
	CMC	0.2490	0.2051	0.1756	0.2724	0.2354	0.2294	0.1351
	DPClus	0.2171	0.1796	0.1339	0.2913	0.2612	0.2473	0.1351
	MCL	0.0974	0.0711	0.0518	0.1196	0.0960	0.1624	2.247e-8
	MCODE	0.1908	0.1472	0.1088	0.2194	0.1853	0.1529	0.1372
	MINE	0.1749	0.1394	0.1168	0.2049	0.1745	0.1415	0.0737
	RNSC	0.2069	0.1776	0.1205	0.2767	0.2554	0.2181	0.1350
	SPICi	0.2431	0.2069	0.1819	0.3179	0.2861	0.3036	**0.1385**

The weights assigned to the original PPI network are presented after “Original”.

As shown in Table 3, for all the four PPI networks, EC-BNMF has competitive performance with other methods in terms of the three measures (Jaccard, PR and *f*-measure) with respect to MIPS and SGD complexes. Furthermore, similar to the results shown in [16], [38], [39], among the ten base clustering algorithms, none of them can dominate other methods on all networks according to the three measures. In particular, on Collins, MCODE consistently outperforms other methods, while RNSC also has a competitive performance. On Gavin, SPICi performs better than other methods with respect to SGD complexes, while CMC and RNSC have good performance with respect to MIPS complexes. On Krogan, CMC outperforms other methods with respect to MIPS complexes, and DPClus outperforms other methods with respect to SGD complexes. On BioGRID, SPICi and ClusterONE output higher quality clusters than others. These results illustrate that different approaches have complimentary strengths. The effectiveness of EC-BNMF in detecting protein complexes is mainly due to its ability of capturing information from multiple clustering results in a unified inference procedure. This is achieved by allocating proper weights to base clustering results and seeking the consistent dense regions among these results to output more accurate and reliable clustering results.

One may have noticed that for some PPI networks such as Collins, the evaluation scores obtained by EC-BNMF are close to some base clustering algorithms. This may be due to the clustering results of the base clustering algorithms are very similar on this PPI network. EC-BNMF is an ensemble algorithm whose performance depends on the base clustering results. Therefore, if the base clustering results are close to each other, the performance improvement may not be very noticeable. However, stability is one of the advantages of EC-BNMF. Even though some methods could obtain similar performance to EC-BNMF with respect to a single measure or a single gold standard, they can not perform well on all PPI networks. For example, compared with other base clustering algorithms, the complexes detected by MCODE are more accurate on Collins, whereas on the other three PPI networks, the complexes detected by MCODE are not very accurate. On Krogan, the complexes detected by CMC are more accurate than other base clustering algorithms with respect to MIPS complexes. When considering SGD complexes, the complexes detected by DPClus are more accurate. But on the other three PPI netoworks, the complexes detected by CMC and DPClus may not well match the known complexes. Furthermore, EC-BNMF allows overlaps between protein complexes. Although some base clustering algorithms perform well on some PPI networks (RNSC performs well on Gavin, and SPICi performs well on Gavin and BioGRID), they can not discover overlapping complexes. Viewed in this light, we provide an alternative method to identify protein complexes, which can discover overlapping complexes and has stable performance on networks with different topological features.

To evaluate the overall performance of each algorithm, we integrate the measurement results of each algorithm on different PPI networks into a final score by weighted combination. The weight of each PPI network indicates the number of proteins in this network, divided by the total number of proteins in all the four PPI networks. Let 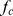, 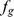, 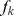 and 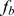 denote the weights of Collins, Gavin, Krogan, and BioGRID respectively. Then we can calculate their value: 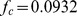, 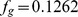, 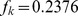 and 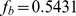. The measurement results of an algorithm on a PPI network can be viewed as a 6-dimensional vector (Jaccard, PR and *f*-measure with respect to MIPS and SGD). The final score of each algorithm is a weighted combination of its measurement results on four PPI networks (e.g., final score of MCL can be computed by 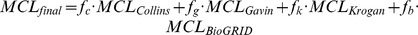. Here, 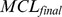, 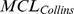, 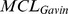, 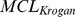 and 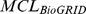 denote the final score of MCL, the measurement results of MCL on Collins, Gavin, Krogan and BioGRID respectively). Note that CFinder is just run on three PPI networks (Collins, Gavin and Krogan), its final score should be calculated according to its performance on these three networks (Here, the weights of these three networks are 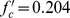, 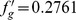 and 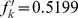 respectively.). To be fair, the average performance of EC-BNMF on these three networks is also calculated. The final scores of different algorithms are listed in Table 4. From Table 4, we can see that EC-BNMF has competitive overall performance with the base clustering algorithms.

**Table 4:** Final scores of different protein complex identification algorithms.

	Algorithm	MIPS			SGD		
		Jaccard	PR	f-measure	Jaccard	PR	f-measure
Final score	EC-BNMF	**0.3484**	**0.3181**	**0.3298**	**0.4286**	**0.4116**	**0.4248**
on four networks	ClusterONE	0.2676	0.2345	0.2281	0.3542	0.3331	0.3644
	COPRA	0.1107	0.1043	0.1262	0.1163	0.1114	0.1749
	CMC	0.2985	0.2553	0.2279	0.3343	0.3073	0.3073
	DPClus	0.2779	0.2442	0.2327	0.3660	0.3413	0.3598
	MCL	0.1857	0.1595	0.1396	0.2254	0.2029	0.2564
	MCODE	0.2449	0.2058	0.1949	0.2850	0.2548	0.2667
	MINE	0.2408	0.2070	0.2034	0.2817	0.2556	0.2758
	RNSC	0.2666	0.2389	0.2042	0.3424	0.3238	0.3208
	SPICi	0.2907	0.2587	0.2556	0.3757	0.3504	0.3924
Final score	EC-BNMF	**0.3814**	**0.3555**	**0.3862**	**0.4779**	**0.4624**	**0.5166**
on three networks	CFinder	0.3265	0.3023	0.2985	0.3945	0.3821	0.3966

Table 3 also lists the values of weights 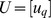 inferred by EC-BNMF for each feature network. From Table 3, we can see that algorithms have better performances always get higher weights, while the poor ones always obtain lower weights. For instance, MCODE has best performance on Collins, thus it obtains the highest weight 0.1450. COPRA performs the worst on Collins, so it gets the lowest weight 6.437e-6 which is close to zero. Therefore, EC-BNMF is able to effectively utilize the information contained in different clustering results. In addition, for all the four PPI networks, we find that COPRA always has poor performance in detecting protein complexes, so the weights assigned to it are always close to zero. These results demonstrate that EC-BNMF is robust when combining different clustering results.

#### Comparison with other ensemble clustering algorithm

**Figure 6:**
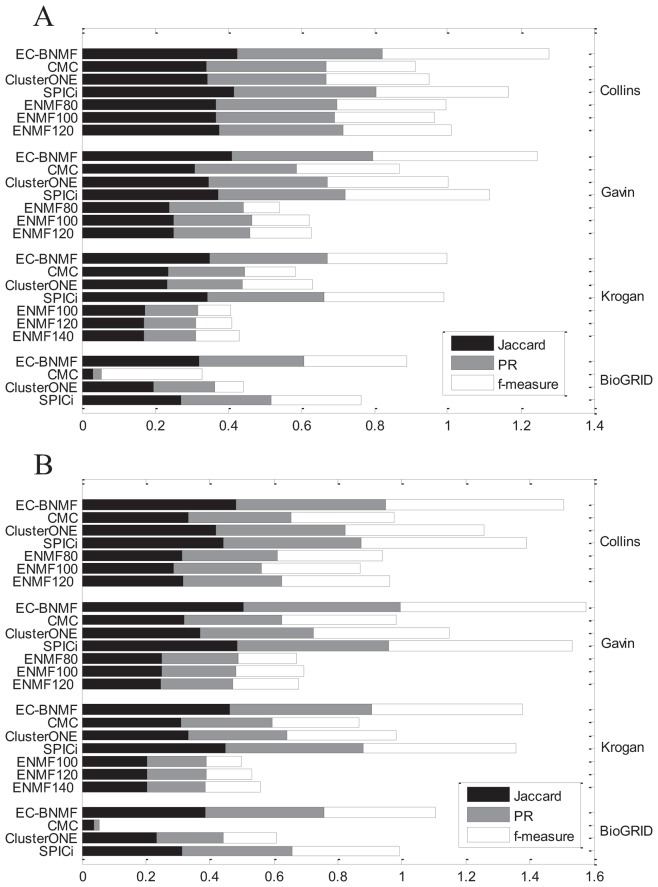
Comparison with other ensemble clustering algorithms. Performance of EC-BNMF in comparison with CMC, ClusterONE, SPICi and Ensemble NMF on four PPI networks in terms of PR, Jaccard and f-measure with respect to (A) MIPS gold standard and (B) SGD gold standard.

In this section, we compare EC-BNMF with Ensemble NMF clustering algorithm [35] which is also developed for clustering PPI networks. For each PPI network, we use the default settings of parameters in the software except two parameters-the range for selecting number of clusters in each factorization and the maximum number 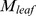 of leaf nodes in final soft hierarchy. For Collins, we set the range to be 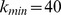 and 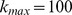. For Gavin, we set 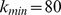 and 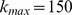. For Krogan, we set 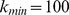 and 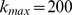. Since the true number of complexes for each network is unknown, and the authors did not clearly mention how to determine the number of complexes in their paper, we use the leaf nodes in final soft hierarchy as clusters. Furthermore, there is not prior information about 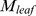, so we select three numbers for each network and test their performance. For Collins and Gavin, we set 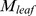 to be 80, 100 and 120. For Krogan, we set 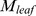 to be 100, 120 and 140. As mentioned in [35], Ensemble NMF clustering algorithm is considerably more computationally complex than standard hierarchical clustering techniques. We do not list its results on BioGRID since it can not give a clustering result in 48 hours. The comparison of these two algorithms are shown in Figure 6. As we have mentioned above, Asur *et al.* [34] also developed an ensemble clustering algorithm to clustering PPI networks. We do not compare EC-BNMF with the ensemble clustering algorithm proposed by Asur *et al.* [34] because there are too many parameters need to be predefined and how to determine their value is not clearly mentioned.

Given a PPI network and a collection of clustering results, after getting the ensemble PPI network, the task is turned into detecting protein complexes from this ensemble PPI network which is a weighted undirected network. Besides Bayesian NMF model, there are several methods can deal with weighted networks such as CMC [51]. In order to illustrate the advantages of EC-BNMF, we design a heuristic comparison. We apply CMC, ClusterONE, and SPICi on the ensemble PPI network and evaluate their performance according to the evaluation criteria proposed above. All of these three algorithms are able to detect complexes from weighted PPI networks directly and output the results in a reasonable time. Other algorithms that can deal with weighted PPI networks are not considered since they can not output the results in a reasonable time. Furthermore, CMC, ClusterONE and SPICi can not automatically update the value of weights *U*, so we apply these three algorithms on the naive ensemble PPI network where 
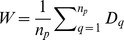
. The results of these three algorithms are obtained over the best tuned parameters. The comparison are shown in Figure 6.

It can be observed from Figure 6 that EC-BNMF has competitive performance with other compared algorithms in terms of the three measures on the four PPI networks. With respect to the performance of SPICi on the original PPI network in Table 3, SPICi has a notable gains in accuracy on the ensemble PPI network, demonstrating that incorporation of diverse feature networks (base clustering results) yields improved performance. EC-BNMF can construct an informative ensemble PPI network and effectively utilize the information contained in this ensemble PPI network, thus it can output more accurate and reliable results. The poor performance of CMC and ClusterONE on ensemble PPI network demonstrates that they may not be well suited to handle such data. As shown in Figure 6, Ensemble NMF clustering algorithm can not output competitive results. Ensemble NMF clustering algorithm only utilizes a single algorithm to produce the base clustering results, thus it could only capture one aspect of the data. Furthermore, if we try the more appropriate parameters, the performance of Ensemble NMF clustering algorithm may be improved, but we have no criteria for selecting parameters.

#### Detecting multi-functional proteins

**Figure 7:**
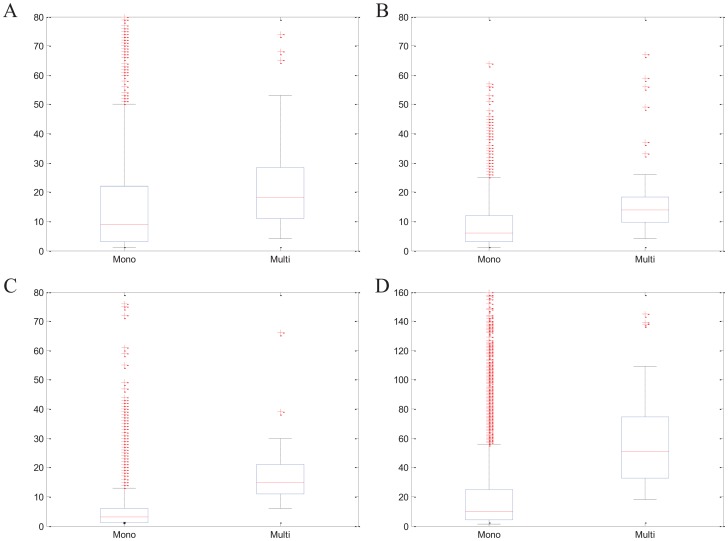
Degree of multi-clustered versus mono-clustered proteins. For degree, the distributions of mono- and multi-clustered proteins are represented by boxplots (line  =  median). (A) Collins network. (B) Gavin network. (C) Krogan network. (D) BioGRID network.

**Figure 8:**
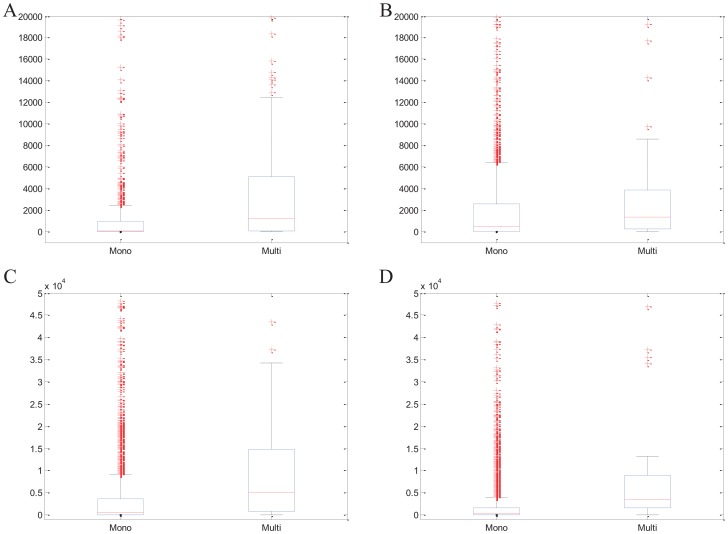
Betweenness of multi-clustered versus mono-clustered proteins. For betweenness, the distributions of mono-and multi-clustered proteins are represented by boxplots (line  =  median). (A) Collins network. (B) Gavin network. (C) Krogan network. (D) BioGRID network.

**Figure 9:**
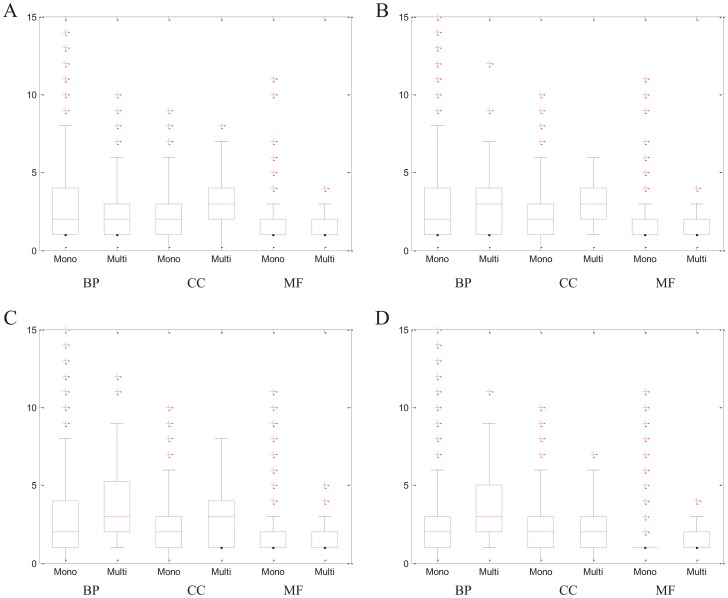
Functional annotations of mono-versus multi-clustered proteins. Quantitative comparison of the number of Gene Ontology terms associated with mono-and multi-clustered proteins. The distributions of mono-and multi-clustered proteins are represented by boxplots (line  =  median). (A) Collins network. (B) Gavin network. (C) Krogan network. (D) BioGRID network.

In fact, some proteins are believed to exhibit different functions while interacting with different partners. Therefore, an approach for protein complex detection should be able to accommodate proteins that are present in more than one complex. As we have mentioned above, EC-BNMF allows a protein to belong to more than one complex. To illustrate the effectiveness of EC-BNMF in detecting multi-functional proteins, we draw support from the functional annotations for the multi-clustered proteins detected by EC-BNMF, with respect to Gene Ontology (GO) database [50]. Complete lists of the functional annotations for the multi-clustered proteins detected by EC-BNMF on four PPI networks can be found in Table S1. Furthermore, similar to [56], we test whether topological and functional features can distinguish multi-clustered proteins from mono-clustered proteins. The corresponding results are shown in Figure 7, Figure 8 and Figure 9.

From Figure 7 and Figure 8, we can find that multi-clustered proteins have, on average, a higher degree and a higher node betweenness, and this is true for all the four PPI networks (Wilcoxon text, for Collins, 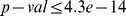 both for degree and betweenness. For Gavin, 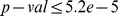 both for degree and betweenness. For Krogan, 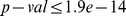 both for degree and betweenness. For BioGRID, 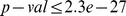 both for degree and betweenness). From Figure 9, we can observed that multi-clustered proteins are, on average, annotated to more GO terms than mono-clustered proteins, in terms of three ontologies (Biological Process, Cellular Component and Molecular Function. For Collins, 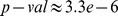 for Cellular Component. For Gavin, 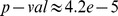 for Cellular Component. For Krogan, 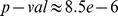 for Cellular Component. For BioGRID, 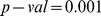 for Cellular Component). For detailed analysis about the Wilcoxon test, please refer to Text S4. Based on the preceding discussion, we find that multi-clustered proteins involved in a larger number of process than mono-clustered proteins. Therefore, EC-BNMF is effective in detecting multi-functional proteins.

**Figure 10:**
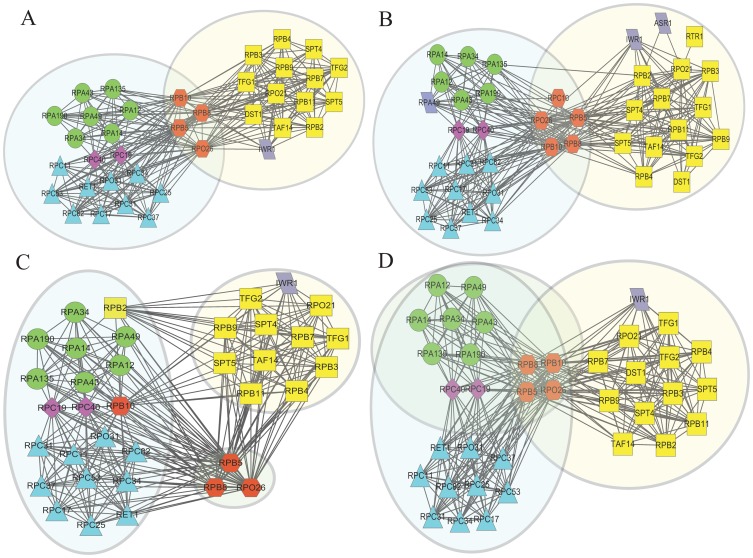
Detecting overlapping complexes. The RNA polymerase I, II, III detected by (A) CFinder. (B) ClusterONE. (C) MCODE. (D) EC-BNMF on Collins. Proteins are labeled according to the complex they belong to: green circle nodes represent RNA polymerase I, yellow rectangle nodes represent RNA polymerase II, blue triangle nodes represent RNA polymerase III and light purple parallelogram nodes represent proteins with other functions. Proteins shared by all the three complexes are labeled with red hexagon, while proteins shared by RNA polymerase I and III are labeled with purple diamond. Shaded areas represent the clusters detected by the corresponding method. This figure is plotted with software Cytoscape [57].

To highlight the advantages of EC-BNMF in detecting multi-functional proteins, we present an illustrative example of how three complexes with known overlaps are detected by CFinder, ClusterONE, MCODE and EC-BNMF in Figure 10 A, B, C and D. For more examples, please refer to Text S5. The clusters shown in Figure 10 are drawn from the clustering results of CFinder, ClusterONE, MCODE and EC-BNMF on Collins. The green circle nodes represent RNA polymerase I; The yellow rectangle nodes represent RNA polymerase II; The blue triangle nodes represent RNA polymerase III and the light purple parallelogram nodes represent proteins with other functions. Shaded areas represent the clusters detected by the corresponding method. It can be observed from Figure 10 A, B and C that CFinder, ClusterONE and MCODE can not correctly detect these three overlapping complexes. Each of them have their own advantages and limitations. In particular, they all cluster RNA polymerase I and III together.

As can be seen from Figure10D, the clusters obtained by EC-BNMF can correctly classify these three complexes. Furthermore, four proteins (YOC224C, YOR210W, YBR154C and YPR187W) common to RNA polymerase I, II and III are correctly classified. Two other proteins (YNL113W and YPR110C) that are shared by RNA polymerase I and III are also correctly classified. This example demonstrates that EC-BNMF can integrate diverse base clustering results into a more accurate and reliable results. One may have noticed that cluster associated with RNA polymerase III contains cluster associated with RNA polymerase I. The reason lies on the following two facts. First, these two complexes share more proteins and have intensive interactions. Second, most of the base clustering results cluster these two complexes together. Thus in the ensemble PPI network, they tend to be of the same cluster. Nevertheless, EC-BNMF can correctly identify the proteins belong to RNA polymerase I and the proteins shared between these three complexes.

## Discussion and Conclusion

The identification of protein complexes will bring richer biological information in gaining insights into the working mechanism of cell and revealing the disease mechanisms. In recent years, numerous mathematical and computer algorithms have been proposed to tackle this problem. Most of these algorithms are designed to explore specific structures in the network. They are based on different optimization criterion and different assumptions of the inner structure of protein complex. Therefore, a single algorithm can only capture one aspect of the PPI network. As Song and Singh [16] mentioned in their study, no single algorithm performs best on all networks. For example, many researchers consider densely connected subgraphs as protein complexes. Under this assumption, protein complexes should be highly connected internally and sparsely connected with the rest of the network. However, this view could not fully describe the characteristic of the PPI network since besides densely connected substructures, complexes with sparsely connected substructures also exist (e.g., linear shape). Furthermore, traditional protein complex identification algorithms that do not support overlap between complexes can not reveal the biological reality. Besides, how to determine the number of complexes in a PPI network is still an open question.

To address these problems, in this study, an alternative method (EC-BNMF) is proposed to identify protein complexes. EC-BNMF is a novel weighted ensemble clustering algorithm which can integrate the clustering results of different protein complex identification algorithms and generate an accurate and reliable clustering result. Unlike conventional ensemble clustering algorithms that treat each base clustering result equally, EC-BNMF is a weighted ensemble clustering algorithm which can automatically estimate the optimal weights of different base clustering results. Therefore, base clustering results that obtain higher weights may be more reliable and can be regarded as important features. On the contrary, base clustering results with lower weights may be less reliable features and they may be far away from real cases. With these weights, we can do selections among features, and output more reliable results. Thus, stability is one of the advantages of EC-BNMF. Experimental results on four yeast PPI networks well verify the stability and effectiveness of EC-BNMF in detecting protein complexes. Further, EC-BNMF allows overlaps between protein complexes, which is closer to the reality.

In fact, as far as we known, two other ensemble clustering algorithms [34], [35] have been developed to detect protein complexes from PPI networks. We do not compare EC-BNMF to Asur method [34] not only for their method need to pregiven the number of complexes which is always unknown, but also for there are many parameters need to be predefined which are not clearly mentioned in their paper and there is no public software available. Hence, we compare our model with Ensemble NMF [35]. Furthermore, to demonstrate the effectiveness of EC-BNMF, we also design some heuristic comparisons. For instance, we apply Bayesian NMF model on the original PPI network and apply CMC, ClusterONE and SPICi on the ensemble PPI network. Anyhow, our analysis show that EC-BNMF can strengthen the quality of simple algorithms, and obtain more accurate results.

As an ensemble clustering algorithm, on the one hand, EC-BNMF has improved performance than individual clustering algorithms, and can alleviate the interference of unreliable clustering results. On the other hand, the performance of EC-BNMF depends on the base clustering results. If all these results are generated by random or computed by poor clustering algorithms, they may far away from real cases. In such a case, the performance of EC-BNMF may also be poor. To alleviate this problem, we also regard the original PPI network as a feature network. Therefore, if most of the base clustering results are unreliable, EC-BNMF can assign higher weight on the original PPI network and assign lower weights on these bad results. In this way, the performance of EC-BNMF is less dependent on the base clustering results. However, a key aspect of EC-BNMF is its ability of integrating multiple features of the PPI network and generating more reliable results. We are concerned with how to get an accurate and informative clustering, therefore, we choose some popular algorithms as base clustering algorithms since their results can effectively describe the network. In addition, the multi-functional proteins discovered by EC-BNMF are also relied on base clustering results. Based on these base clustering results, EC-BNMF can filter out unreliable multi-functional proteins and add in more reliable multi-functional proteins.

We now count the overall time cost of the updating process in Equation (8), (9) and (10). The time cost for updating *H* is 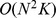, where *N* is the number of proteins, and *K* is the number of complexes. The time cost for updating 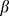 is 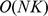 and the time cost for updating *U* is 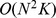. Therefore the overall time cost of EC-BNMF is 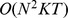, where *T* is the number of iterations. Since the parameter *H* is sparse, the real time cost is much smaller than 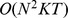. In addition, before performing our ensemble algorithm, we need to compute 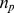 base clustering results, which is time consuming. Nevertheless, this research is still meaningful for the following reasons: First, as mentioned in [35], in the context of understanding and exploiting the structure in PPI networks, cluster analysis is used as an “offline” process, where producing an accurate and reliable clustering is the primary goal. Second, when generating base clustering results through some protein complex identification algorithms, we can use the softwares provided by the authors to implement these algorithms, which are written in C++ language or Java. Third, with the rapid development of computer hardware, we have the ability to undertake large amount of operations. Fourth, the generation process of base clustering results can be parallelized. Therefore, by parallel computing, we can generate different clustering results simultaneously on modern multi-core processors and reduce the running times.

In this paper, we use Poisson distribution, Half-Normal distribution and inverse-Gamma distribution to model the generation process of the ensemble PPI network. Indeed, other distributions such as Bernoulli distribution, binomial distribution and exponential distribution can also be tried. As an ensemble clustering algorithm, our model is more flexible. It is of great interest to use this model to undertake other clustering-based tasks such as exploring modules in gene regulatory networks and cell signaling networks.

## Supporting Information

Table S1 **Complete lists of the functional annotations for the multi-clustered proteins detected by EC-BNMF on the four PPI networks.** For each protein, we present the GO terms associated with it, in terms of three ontologies (Biological Process, Cellular Component and Molecular Function).
(XLS)

Text S1 **Detailed inference of the solution to Bayesian NMF-based weighted Ensemble Clustering.**
(PDF)

Text S2 **The three metrics used for evaluating the predicted protein complexes.**
(PDF)

Text S3 **Brief description and detailed parameter settings of the base clustering algorithms.**
(PDF)

Text S4 **Comparison of the number of Gene Ontology annotations between mono-clustered and multi-clustered proteins.**
(PDF)

Text S5 **More examples of overlapping protein complexes detected by base clustering algorithms.**
(PDF)
