# Supplementary material for: Protein Complex Detection via Weighted Ensemble Clustering Based on Bayesian Nonnegative Matrix Factorization
Source: PLoS One. 2013 May 2;8(5):e62158. doi: 10.1371/journal.pone.0062158 (PMC3642239; doi:10.1371/journal.pone.0062158)
Supplement: Text S1 — Detailed inference of the solution to Bayesian NMF-based weighted Ensemble Clustering. (PDF) [file pone.0062158.s002.pdf]

Solution to Bayesian Nonnegative Matrix Factorization-based weighted Ensemble Clustering

Le Ou-Yang, Dao-Qing Dai, and Xiao-Fei Zhang

The objective function of Bayesian Nonnegative Matrix Factorization-based weighted Ensemble Clustering (EC-BNMF):

$$\begin{aligned}\min_{U,H,\beta} J(U,H,\beta) &= -\log P(W,H,\beta) + \lambda \sum_{q=1}^{n_p} u_q \log(u_q) \\ &= -\sum_{i=1}^{N}\sum_{j=1}^{N}\Big((\sum_{q=1}^{n_p} u_q (D_q)_{i,j}) \log(HH^T)_{i,j} - (HH^T)_{i,j}\Big) \\ &\quad + \sum_{i=1}^{N}\sum_{z=1}^{K} \frac{1}{2\beta_z}(h_{i,z})^2 + \frac{N}{2}\sum_{z=1}^{K}\log\beta_z + \sum_{z=1}^{K}\frac{b}{\beta_z} \\ &\quad + (a+1)\sum_{z=1}^{K}\log\beta_z + \lambda\sum_{q=1}^{n_p} u_q\log(u_q). \qquad (1) \\ s.t. \qquad & H \geq 0, \text{ and } \sum_{q=1}^{n_p} u_q = 1,\ u_q \geq 0, \text{ for } q = 1,\cdots,n_p.\end{aligned}$$

Here coefficient $\lambda \geq 0$ is the tradeoff parameter for controlling the contribution of the regularization term.

Given U, (1) degenerates to the following objective function:

$$\begin{aligned}\min_{H,\beta} J(H,\beta) &= -\log P(W,H,\beta) \\ &= -\sum_{i=1}^{N}\sum_{j=1}^{N}\Big((\sum_{q=1}^{n_p} u_q (D_q)_{i,j}) \log(HH^T)_{i,j} - (HH^T)_{i,j}\Big) \\ &\quad + \sum_{i=1}^{N}\sum_{z=1}^{K} \frac{1}{2\beta_z}(h_{i,z})^2 + \frac{N}{2}\sum_{z=1}^{K}\log\beta_z + \sum_{z=1}^{K}\frac{b}{\beta_z} \qquad (2) \\ &\quad + (a+1)\sum_{z=1}^{K}\log\beta_z, \\ s.t. \qquad & H \geq 0.\end{aligned}$$

Thus we minimize $J(H, \beta)$ with respect to H and β. To solve the constrained optimization problem (2), we adopt the multiplicative update rules [3, 4]. Let $\phi_{i,z}$ be the Lagrange multiplier for constraint $h_{i,z} \geq 0$, and $\Phi = (\phi_{i,z})$. The Lagrange function $\mathcal{L}$ is:

$$\begin{aligned}\mathcal{L}(H, \beta, \Phi) \quad = \quad & -\sum_{i=1}^{N}\sum_{j=1}^{N}\Big((\sum_{q=1}^{n_p} u_q (D_q)_{i,j}) \log(HH^T)_{i,j} - (HH^T)_{i,j}\Big) \\ & +\sum_{i=1}^{N}\sum_{z=1}^{K}\frac{1}{2\beta_z}(h_{i,z})^2 + \frac{N}{2}\sum_{z=1}^{K}\log\beta_z + \sum_{z=1}^{K}\frac{b}{\beta_z} \\ & +(a+1)\sum_{z=1}^{K}\log\beta_z + \sum_{i=1}^{N}\sum_{z=1}^{K}\phi_{i,z}h_{i,z}.\end{aligned} \quad (3)$$

We can find the gradient of the Lagrange function $\mathcal{L}(H, \beta, \Phi)$ with respect to $h_{i,l}$ and β_l:

$$\nabla_{h_{i,l}}\mathcal{L}(H, \beta, \Phi) = -2\sum_{j=1}^{N}(\sum_{q=1}^{n_p} u_q (D_q)_{i,j})\frac{h_{j,l}}{\sum_{z=1}^{K} h_{i,z}h_{j,z}} + 2\sum_{j=1}^{N} h_{j,l} + \frac{1}{\beta_l}h_{i,l} + \phi_{i,l}.$$

and

$$\nabla_{\beta_l}\mathcal{L}(H, \beta, \Phi) = -\frac{1}{2}\frac{1}{\beta_l^2}\sum_{i=1}^{N} h_{i,l}^2 + \frac{N}{2}\frac{1}{\beta_l} - b\frac{1}{\beta_l^2} + (a+1)\frac{1}{\beta_l}.$$

Since the estimators of $h_{i,l}$ and β_l need to satisfy $\nabla_{h_{i,l}}\mathcal{L} = 0$ and $\nabla_{\beta_l}\mathcal{L} = 0$, we can obtain:

$$\phi_{i,l} = 2\sum_{j=1}^{N}(\sum_{q=1}^{n_p} u_q (D_q)_{i,j})\frac{h_{j,l}}{\sum_{z=1}^{K} h_{i,z}h_{j,z}} - 2\sum_{j=1}^{N} h_{j,l} - \frac{1}{\beta_l}h_{i,l}. \quad (4)$$

and

$$\beta_l = \frac{2b + \sum_{i=1}^{N} h_{i,l}^2}{N + 2a + 2}. \quad (5)$$

According to the Karush-Kuhn-Tucker (KKT) conditions [2], $\phi_{i,l}h_{i,l} = 0$, we obtain the following equation for $h_{i,l}$:

$$h_{i,l}\left(2\sum_{j=1}^{N}(\sum_{q=1}^{n_p} u_q (D_q)_{i,j})\frac{h_{j,l}}{\sum_{z=1}^{K} h_{i,z}h_{j,z}}\right) = h_{i,l}\left(2\sum_{j=1}^{N} h_{j,l} + \frac{1}{\beta_l}h_{i,l}\right). \quad (6)$$

Therefore, we obtain the updating rule for $h_{i,l}$:

$$h_{i,l} \leftarrow h_{i,l}\frac{\sum_{j=1}^{N}\frac{(\sum_{q=1}^{n_p} u_q (D_q)_{i,j})h_{j,l}}{\sum_{z=1}^{K} h_{i,z}h_{j,z}}}{\sum_{j=1}^{N} h_{j,l} + \frac{1}{2\beta_l}h_{i,l}}. \quad (7)$$

Ding et al.[1] suggest that in practice, the following variant of (7) works better:

$$h_{i,l} \leftarrow \frac{h_{i,l}}{2} + \frac{1}{2} h_{i,l} \frac{\sum_{j=1}^{N} \frac{(\sum_{q=1}^{n_p} u_q (D_q)_{i,j}) h_{j,l}}{\sum_{z=1}^{K} h_{i,z} h_{j,z}}}{\sum_{j=1}^{N} h_{j,l} + \frac{1}{2\beta_l} h_{i,l}}. \quad (8)$$

Through Equation (5), we obtain the updating rule for β_l:

$$\beta_l \leftarrow \frac{2b + \sum_{i=1}^{N} h_{i,l}^2}{N + 2a + 2}. \quad (9)$$

By the multiplicative update rules, if we initialize $h_{i,z}$ with nonnegative value, the value of $h_{i,z}$ will remain nonnegative.
After an update of H and β, we fix their values, and (1) degenerates to the following constrained optimization problem:

$$\begin{aligned} \min_U J_U(U) &= -\sum_{i=1}^{N} \sum_{j=1}^{N} (\sum_{q=1}^{n_p} u_q (D_q)_{i,j}) \log(HH^T)_{i,j} + \lambda \sum_{q=1}^{n_p} u_q \log(u_q). \\ s.t. \quad & \sum_{q=1}^{n_p} u_q = 1. \end{aligned} \quad (10)$$

We use the Lagrangian multiplier technique to solve the following unconstrained minimization problem:

$$\begin{aligned} \mathcal{L}(U, \gamma) &= -\sum_{i=1}^{N} \sum_{j=1}^{N} (\sum_{q=1}^{n_p} u_q (D_q)_{i,j}) \log(HH^T)_{i,j} \\ &+ \lambda \sum_{q=1}^{n_p} u_q \log(u_q) + \gamma (\sum_{q=1}^{n_p} u_q - 1). \end{aligned} \quad (11)$$

Here γ is the Lagrangian multiplier for constrain $\sum_{q=1}^{n_p} u_q = 1$. To find the minimizer of $\mathcal{L}(U, \gamma)$, we vanish the gradients in all variables. Thus:

$$\frac{\partial \mathcal{L}(U, \gamma)}{\partial u_m} = -\sum_{i=1}^{N} \sum_{j=1}^{N} (D_m)_{i,j} \log(HH^T)_{i,j} + \lambda(\log u_m + 1) + \gamma = 0 \quad (12)$$

and

$$\frac{\partial \mathcal{L}(U, \gamma)}{\partial \gamma} = \sum_{q=1}^{n_p} u_q - 1 = 0. \quad (13)$$

From (12), we have

$$u_m = \exp(\frac{1}{\lambda} \sum_{i=1}^{N} \sum_{j=1}^{N} (D_m)_{i,j} \log(HH^T)_{i,j}) \exp(-1) \exp(-\frac{\gamma}{\lambda}) \quad (14)$$

Substituting (14) into (13), we get

$$u_m = \frac{exp(\frac{1}{\lambda}\sum_{i=1}^{N}\sum_{j=1}^{N}(D_m)_{i,j}\log(HH^T)_{i,j})}{\sum_{q=1}^{n_p} exp(\frac{1}{\lambda}\sum_{i=1}^{N}\sum_{j=1}^{N}(D_q)_{i,j}\log(HH^T)_{i,j})} \tag{15}$$

By initializing H randomly and updating H, β and U iteratively according to Equations (8), (9) and (15) respectively, we can obtain the solution to the Bayesian NMF-based weighted Ensemble Clustering (1).

References

[1] C. Ding, X. He, and H.D. Simon. On the equivalence of nonnegative matrix factorization and spectral clustering. In *Proc. SIAM Data Mining Conf*, number 4, pages 606–610, 2005.

[2] H.W. Kuhn and A.W. Tucker. Nonlinear programming. In *Proceedings of the second Berkeley symposium on mathematical statistics and probability*, volume 1, pages 481–492. California, 1951.

[3] Daniel D. Lee and H. Sebastian Seung. Algorithms for non-negative matrix factorization. In *Advances in neural information processing systems*, volume 13, pages 556–562, 2001.

[4] D. Seung and L. Lee. Algorithms for non-negative matrix factorization. volume 13, pages 556–562, 2001.
