# Supplementary material for: Protein Complex Detection via Weighted Ensemble Clustering Based on Bayesian Nonnegative Matrix Factorization
Source: PLoS One. 2013 May 2;8(5):e62158. doi: 10.1371/journal.pone.0062158 (PMC3642239; doi:10.1371/journal.pone.0062158)
Supplement: Text S2 — The three metrics used for evaluating the predicted protein complexes. (PDF) [file pone.0062158.s003.pdf]

Evaluation criteria

Le Ou-Yang, Dao-Qing Dai, and Xiao-Fei Zhang

The quality of complexes detected by computational algorithms are evaluated based on the following three scoring measures. The first one is f-measure which is defined as the harmonic mean of Precision and Recall [1]. The other two are proposed by Song and Singh [2]. Among these three measures, f-measure is used to assess the similarity between predicted complexes and reference complexes at complex level (Recall measures what fraction of the reference sets are matched by the predicted complexes, and Precision measures what fraction of the predicted complexes are matched by the reference complexes). Jaccard and PR metrics can measure how well the predicted complexes correspond to reference complexes at complex-protein pair level, which take into account the number of proteins in each complex. The value of each measure vary between 0 and 1, and the higher value means better overlaps.

We first give some notations before describing these measures. Let P denote the number of complexes detected by a particular algorithm and T denote the number of reference complexes. Let C_i represents the set of proteins belong to the i-th detected complex and G_j represents the set of proteins belong to the j-th reference complex.

We say a detected complex C_i and a reference complex G_j match each other if:

$$\frac{|C_i \bigcap G_j|}{|C_i|} > \delta \ and \ \frac{|C_i \bigcap G_j|}{|G_j|} > \delta. \tag{1}$$

where δ is an input parameter between 0 and 1 which we set to 0.5 in this study. Given a set of predicted complexes $\mathcal{C} = \{C_1, C_2, \cdots, C_P\}$ and a set of reference complexes $\mathcal{G} = \{G_1, G_2, \cdots, G_T\}$, Precision and Recall are defined as follows:

$$Precision = \frac{|\{C_i | C_i \in \mathcal{C} \wedge \exists G_j \in \mathcal{G}, G_j \ matches \ C_i\}|}{P}, \tag{2}$$

$$Recall = \frac{|\{G_j | G_j \in \mathcal{G} \wedge \exists C_i \in \mathcal{C}, C_i \ matches \ G_j\}|}{T}. \tag{3}$$

In order to take into account of both the Precision and Recall, an integrated method called f-measure is used.

$$f - measure = \frac{2 \times Precision \times Recall}{Precision + Recall}. \tag{4}$$

The other two measures are defined as follows:

Jaccard measure: let $Jac_{i,j} = \frac{|C_i \bigcap G_j|}{|C_i \bigcup G_j|}$ represent the Jaccard coefficient between detected complex C_i and reference complex G_j. For each detected complex C_i, we find the reference complex that maximizes the Jaccard coefficient between them, which is

defined as $JaccardC_i = \max_j Jac_{i,j}$. Similarly, for each reference complex G_j, we try to find the detected complex that maximizes the Jaccard coefficient between them, that is $JaccardG_j = \max_i Jac_{i,j}$.

PR measure: The precision-recall (PR)-based score $PR_{i,j}$ between a detected complex C_i and a reference complex G_j is calculated by $PR_{i,j} = \frac{|C_i \bigcap G_j|}{|C_i|} \times \frac{|C_i \bigcap G_j|}{|G_j|}$. The first part $\frac{|C_i \bigcap G_j|}{|C_i|}$ is the precision metric which measures what fraction of the proteins in detected complex C_i correspond to reference complex G_j, and the second part $\frac{|C_i \bigcap G_j|}{|G_j|}$ is the recall metric which measures how much of reference complex G_j is recovered by detected complex C_i. Similar to the definition of ***Jaccard*** measure, we define $PRC_i = \max_j PR_{i,j}$ and $PRG_j = \max_i PR_{i,j}$ for the ***PR*** measure.

For each measure, taking average over all the detected complexes, weighted by the size of each detected complex, we obtain $JaccardC$ and PRC as follows:

$$JaccardC = \frac{\sum_{i=1}^{P} |C_i| \cdot JaccardC_i}{\sum_{i=1}^{P} |C_i|}, \tag{5}$$

and

$$PRC = \frac{\sum_{i=1}^{P} |C_i| \cdot PRC_i}{\sum_{i=1}^{P} |C_i|}. \tag{6}$$

Similarly, the measures $JaccardG$ and PRG for the T reference complexes is $JaccardG = \frac{\sum_{j=1}^{T} |G_j| JaccardG_j}{\sum_{j=1}^{T} |G_j|}$ and $PRG = \frac{\sum_{j=1}^{T} |G_j| \cdot PRG_j}{\sum_{j=1}^{T} |G_j|}$. Finally, we use $Jaccard$ which is the harmonic mean of $JaccardC$ and $JaccardG$, and PR which is the harmonic mean of PRC and PRG to quantify the accuracy of the detected complexes:

$$Jaccard = \frac{2 \times JaccardC \times JaccardG}{JaccardC + JaccardG}, \tag{7}$$

and

$$PR = \frac{2 \times PRC \times PRG}{PRC + PRG}. \tag{8}$$

References

[1] Y. Qi, F. Balem, et al. Protein complex identification by supervised graph local clustering. *Bioinformatics*, 24(13):i250–i258, 2008.

[2] J. Song and M. Singh. How and when should interactome-derived clusters be used to predict functional modules and protein function? *Bioinformatics*, 25(23):3143–3150, 2009.
