# Supplementary material for: Protein Complex Detection via Weighted Ensemble Clustering Based on Bayesian Nonnegative Matrix Factorization
Source: PLoS One. 2013 May 2;8(5):e62158. doi: 10.1371/journal.pone.0062158 (PMC3642239; doi:10.1371/journal.pone.0062158)
Supplement: Text S3 — Brief description and detailed parameter settings of the base clustering algorithms. (PDF) [file pone.0062158.s004.pdf]

Brief description of the base clustering algorithms

Le Ou-Yang, Dao-Qing Dai, and Xiao-Fei Zhang

In this paper, we choose ten state-of-the-art protein complex identification algorithms as base clustering algorithm: CFinder [1], CMC [8], ClusterONE [9], COPRA [5], DPClus [2], MCL [4], MCODE [3], MINE [12], RNSC [7], and SPICi [6]. Table 1 lists the websites where we download the softwares of these algorithms, the version numbers of these softwares and several indications about whether these algorithms support overlapping among complexes and whether they could be applied to weighted PPI networks. Given a PPI network, the performance of each algorithm depends on the choice of the parameters. Therefore, for all the considered algorithms, we set the corresponding parameters to yield the best clustering results. To avoid evaluation bias, while we are selecting parameters, we only consider clusters with at least three components. Furthermore, we consider the following three criterions:

- Three scoring measures (JaccardPR and f-measure) are used to evaluate the performance of each algorithm.
- Two different reference sets (the MIPS complexes and the SGD complexes) are used as gold standards.
- For each algorithm, similar to EC-BNMF, the performance is measured by the harmonic mean of six scores—Jaccrad, PR and f-measure with respect to MIPS and SGD complexes. The final results are obtained by choosing the parameters that yield the best performance.

We briefly review the main features of these algorithms and the setting of parameters for each algorithm in the following.

Table 1: Characteristics of the base clustering algorithms

Algorithm	Downloading website	Version	Overlapping clusters supported	weighted graphs supported
CFinder	http://cfinder.org/	2.0.5	√	√
CMC	http://www.comp.nus.edu.sg/~wongls/projects/complexprediction/CMC-26may09/	2.0	√	√
ClusterONE	http://www.paccanarolab.org/cluster-one/index.html	0.94	√	√
COPRA	http://www.cs.bris.ac.uk/~steve/networks/copra/	-	√	√
DPClus	http://kanaya.naist.jp/DPClus/	-	√	
MCL	http://micans.org/mcl/	09-308		√
MCODE	http://baderlab.org/Software/MCODE	1.32	√	
MINE	http://www.biomedcentral.com/1471-2105/12/192	1.5	√	
RNSC	http://www.cs.utoronto.ca/~juris/data/rnsc/	-		
SPICi	http://compbio.cs.princeton.edu/spici/	-		√

Table 2: Parameters selected for CFinder

Network	Collins	Gavin	Krogan	BioGRID
k-clique size	8	5	5	N/A

N/A for BioGRID network indicates that CFinder can not give any results within 48 hours.

Table 3: Parameters selected for CMC

Network	Collins	Gavin	Krogan	BioGRID
Overlap threshold	0.6	0.6	0.2	0.2
Merging threshold	0.3	0.5	0.5	0.9

CFinder

Palla *et al.* [10] proposed a Clique Percolation Method (CPM) to uncover the overlapping community structure of complex networks. CPM detects overlapping clusters by finding k-clique percolation communities. Here, a k-clique is a complete subgraphs with k nodes and two k-cliques are adjacent if they share $(k-1)$ common nodes. Base on this method, Adamcsek *et al.* [1] provided a software called CFinder to detect overlapping modules in biological networks. Therefore, the performance of CFinder is determined by the size of k-clique, in this paper, for each PPI network, k is taking a value from 3 to 10, step size by 1. Table 2 lists the optimal values of parameter k for each PPI network.

CMC

Liu *et al.* [8] proposed a Clustering algorithm based on Maximal Cliques (CMC) to detect overlapping protein complexes. CMC first finds out all the maximal cliques in PPI networks, then assigns each interaction a score based on a reliability measure. Finally, CMC removes or merges highly overlapping cliques based on their connectivity. Therefore, CMC is primarily governed by the overlap threshold and merging threshold. In this paper, the value of the overlap threshold is from 0.2 to 0.8, with a step size of 0.1, while the value of the merging threshold is from 0 to 1, with a step size of 0.1. The minimum size of the detected complex is set to be 3. Table 3 lists the optimal overlap threshold and merging threshold for each PPI network.

ClusterONE

Nepusz *et al.* [9] recently proposed an algorithm (ClusterONE) to detect overlapping protein complexes in PPI networks. ClusterONE depends on overlapping neighborhood expansion. As suggested by the authors, for all the four PPI networks we use the default settings of parameters in the software.

Table 4: Parameters selected for DPClus

Network	Collins	Gavin	Krogan	BioGRID
d_{in}	0.7	0.7	0.7	0.8
cp_{in}	0.5	0.5	0.5	0.5

Table 5: Parameters selected for MCL

Network	Collins	Gavin	Krogan	BioGRID
Inflation	2.8	3.2	1.8	3.4

COPRA

Gregory [5] developed an algorithm COPRA for finding overlapping community structure in large networks. COPRA is based on the label propagation technique (RAK) proposed by Raghavan *et al.* [11], but is able to detect overlapping communities. In RAK algorithm, each node is first given a unique label, then, repeatedly, each node updates its label by replacing it by the label used by the greatest number of neighbours. Finally, all nodes with the same label are clustered together. To find overlapping communities, COPRA allows a node label to contain more than one community identifier. It brings in a belonging coefficient to indicate the strength of a node's membership of a community, then a parameter is introduced to control the potential degree of overlap between communities. Here, for all the four PPI networks, we use the default settings of parameters in the software.

DPClus

Altaf-Ul-Amin *et al.* [2] proposed a cluster periphery-tracking algorithm (DPClus) to mine dense subgraphs. DPClus weights all the nodes in its first step. Then DPClus takes the highest weighted node as the initial cluster and extends this cluster by adding nodes from its neighbors. DPClus uses two parameters d_{in} and cp_{in} (d_{in} is a value of minimum density and cp_{in} is a minimum value for cluster property) to determine whether a neighbor should be added to the cluster. Here, the values of d_{in} and cp_{in} are ranged from 0.5 to 0.8 with 0.1 as the step size. We list the optimal combination of the values of these parameters for each PPI network in Table 4.

MCL

Markov Clustering Algorithm (MCL) [4] can detect protein complexes by simulating random walks on networks. MCL manipulates the adjacency matrix of a network with two operators called expansion and inflation. The key parameter of MCL is inflation, which tunes the granularity of clustering. Here, we try different values of inflation, ranges from 1.2 to 5.0 with 0.2 increment. The optimal value of inflation for each PPI network is shown in Table 5.

Table 6: Parameters selected for MCODE

Network	Collins	Gavin	Krogan	BioGRID
Depth limit	3	3	3	3
Node score cutoff	0.2	0.4	0.5	0.1
Haircut	on	on	on	on
Fluffing	off	off	off	off
Node density cutoff	N/A	N/A	N/A	N/A

MCODE

MCODE [3] is one of the first computational methods to detect protein complexes, which consists of three stages: vertex weighting, complex prediction and optionally post-processing. In the first stage, MCODE weights all the nodes based on their local neighborhood densities. In the second stage, nodes with high weights are selected as the seed nodes of initial clusters, then MCODE augments these clusters by outward traversing from the seeds. In the post-processing step, MCODE filters out non-dense subgraphs and adds proteins based on connectivity criteria. The depth limit parameter controls the duration of the augment process. The node score cutoff parameter controls the difference that can be tolerance between scores of proteins within the same complex, and it closely related to the size of the complex. There are two possible post-processing operations: haircut and fluffing. MCODE is able to produce overlapping complexes in the fluffing case, but we experimentally find that when fluffing is turned off, MCODE always has better performance. We try all possible combinations of the following parameters:

- Depth limit: 3, 4, 5
- Node score cutoff: 0.1 to 1.0 with a step size of 0.1
- Haircut: on or off
- Fluffing: on or off
- Node density cutoff: 0, 0.1, 0.2

We list the optimal parameters of MCODE for each PPI network in Table 6.

MINE

MINE [12] is an agglomerative clustering method that can identify highly modular sets of proteins within highly interconnected PPI networks. In this paper, we try different value of node score cutoff (from 0.1 to 1 with 0.1 as the step size) and 3 values of depth limit (3, 4, 5). For the other parameters, without stating, we use the default values in the software. The optimal values of the parameters of MINE for each PPI network are listed in Table 7.

Table 7: Parameters selected for MINE

Network	Collins	Gavin	Krogan	BioGRID
Depth limit	3	3	3	3
Node score cutoff	0.1	0.1	0.1	0.1

Table 8: Parameters selected for SPICi

Network	Collins	Gavin	Krogan	BioGRID
Density	0.7	0.7	0.7	0.8

RNSC

King *et al.* [7] proposed a Restricted Neighborhood Search Clustering (RNSC) algorithm to explore the best partition of a network by using a cost function. RNSC starts with a randomly partition of a network, and iteratively moves a node from one cluster to another to decrease the value of cost function. For all the four PPI networks, we use the default settings of parameters in the software.

SPICi

SPICi [6] is a computationally efficient local network clustering algorithm which can be used to detect protein complexes from PPI networks. SPICi seeds clusters with nodes according to their weighted degree, an unclustered node is then added to a cluster if the support is high enough and the density of the cluster remains higher than a user-defined threshold, otherwise, the cluster is output and the nodes in this cluster are removed from the network. SPICi thus has two parameters: the density threshold and the support threshold. Here, we try different values of density threshold, ranges from 0.1 to 1 with 0.1 increment. For the other parameters, we use the default settings in the software. Table 8 lists the optimal value of density parameter for each PPI networks.

References

[1] B. Adamcsek, G. Palla, I.J. Farkas, I. Derényi, and T. Vicsek. Cfinder: locating cliques and overlapping modules in biological networks. *Bioinformatics*, 22(8):1021 – 1023, 2006.

[2] M. Altaf-Ul-Amin, Y. Shinbo, K. Mihara, K. Kurokawa, and S. Kanaya. Development and implementation of an algorithm for detection of protein complexes in large interaction networks. *BMC Bioinformatics*, 7(1):207, 2006.

[3] Gary D Bader and Christopher WV Hogue. An automated method for finding molecular complexes in large protein interaction networks. *BMC Bioinformatics*, 4(1):2, 2003.

[4] A.J. Enright, S. Van Dongen, and C.A. Ouzounis. An efficient algorithm for large-scale detection of protein families. *Nucleic Acids Research*, 30(7):1575–1584, 2002.

[5] S. Gregory. Finding overlapping communities in networks by label propagation. *New Journal of Physics*, 12(10):103018, 2010.

[6] Peng Jiang and Mona Singh. Spici: a fast clustering algorithm for large biological networks. *Bioinformatics*, 26(8):1105 – 1111, 2010.

[7] AD King, N. Pržulj, and I. Jurisica. Protein complex prediction via cost-based clustering. *Bioinformatics*, 20(17):3013–3020, 2004.

[8] G. Liu, L. Wong, and H.N. Chua. Complex discovery from weighted ppi networks. *Bioinformatics*, 25(15):1891 – 1897, 2009.

[9] T. Nepusz, H. Yu, and A. Paccanaro. Detecting overlapping protein complexes in protein-protein interaction networks. *Nature Methods*, 9(5):471–472, 2012.

[10] Gergely Palla, Imre Derenyi, Illes Farkas, and Tamas Vicsek. Uncovering the overlapping community structure of complex networks in nature and society. *Nature*, 435(7043):814–818, 2005.

[11] U.N. Raghavan, R. Albert, and S. Kumara. Near linear time algorithm to detect community structures in large-scale networks. *Physical Review E*, 76(3):036106, 2007.

[12] Kahn Rhrissorrakrai and Kristin C Gunsalus. Mine: module identification in networks. *BMC Bioinformatics*, 12(1):192, 2011.
