# Supplementary material for: Protein Complex Detection via Weighted Ensemble Clustering Based on Bayesian Nonnegative Matrix Factorization
Source: PLoS One. 2013 May 2;8(5):e62158. doi: 10.1371/journal.pone.0062158 (PMC3642239; doi:10.1371/journal.pone.0062158)
Supplement: Text S4 — Comparison of the number of Gene Ontology annotations between mono-clustered and multi-clustered proteins. (PDF) [file pone.0062158.s005.pdf]

Comparison of the number of Gene Ontology annotations between mono-clustered and multi-clustered proteins

Le Ou-Yang, Dao-Qing Dai, and Xiao-Fei Zhang

In the manuscript, we have discussed whether topological and functional features can distinguish multi-clustered proteins from mono-clustered proteins. To further validate these results, we performs a two-sided rank sum test of the null hypothesis that the topological and functional features of multi- and mono-clustered proteins are independent samples from identical continuous distributions with equal medians, against the alternative that they do not have equal medians. The results of the Wilcoxon test for the topological features are listed in the manuscript. To show that multi-clustered proteins contain more functional features than mono-clustered proteins, we draw support from the Gene Ontology (GO) database [1]. This is reflected by the number of GO terms annotating multi-clustered proteins. Through the Wilcoxon tests, we find that multi-clustered proteins are, on average, annotated to more terms than mono-clustered proteins, in terms of all three GO ontologies (Biological Process, Cellular Component and Molecular Function). The results of the Wilcoxon test for Biological Process, Cellular Component and Molecular Function are listed in Table 1.

Table 1: **Comparison of the number of Gene Ontology annotations between mono- and multi-clustered proteins.**

Ontology	Collins	Gavin	Krogan	BioGRID
Biological Process	0.6	0.26	3.6e-6	2.3e-11
Cellular Component	3.3e-6	4.2e-5	8.5e-6	0.001
Molecular Function	0.05	0.0046	2.7e-5	3.4e-10

From Table 1, we can observed that for Collins and Gavin, the Wilcoxon test for Biological Process is not very significant. These may be due to the incompleteness of the Gene Ontology annotations. However, the multi-clustered proteins detected by EC-BNMF have higher degree and higher betweenness than mono-clustered proteins. That means the topological features of multi-clustered proteins are different from mono-clustered proteins. Therefore, if the topological structure of the PPI network is reliable, the multi-clustered proteins detected by EC-BNMF are most likely to be real multi-functional proteins. In this way, EC-BNMF can help predict the functions of proteins. Nevertheless, in most cases, multi-clustered proteins are annotated to more terms than mono-clustered proteins. Thus EC-BNMF is effective in detecting multi-functional

proteins.

References

[1] M. Ashburner, C.A. Ball, J.A. Blake, D. Botstein, H. Butler, J.M. Cherry, A.P. Davis, K. Dolinski, S.S. Dwight, J.T. Eppig, HarrisM A., D P. Hill, L. Issel-Tarver, A. Kasarskis, S. Lewis, J C. Matese, J E. Richardson, M. Ringwald, G M. Rubin, and G. Sherlock. Gene ontology: tool for the unification of biology. *Nat Genet*, 25(1):25–29, 2000.
