# Supplementary material for: Protein Complex Detection via Weighted Ensemble Clustering Based on Bayesian Nonnegative Matrix Factorization
Source: PLoS One. 2013 May 2;8(5):e62158. doi: 10.1371/journal.pone.0062158 (PMC3642239; doi:10.1371/journal.pone.0062158)
Supplement: Text S5 — More examples of overlapping protein complexes detected by base clustering algorithms. (PDF) [file pone.0062158.s006.pdf]

Examples of overlapping protein complexes detected by base clustering algorithms

Le Ou-Yang, Dao-Qing Dai, and Xiao-Fei Zhang

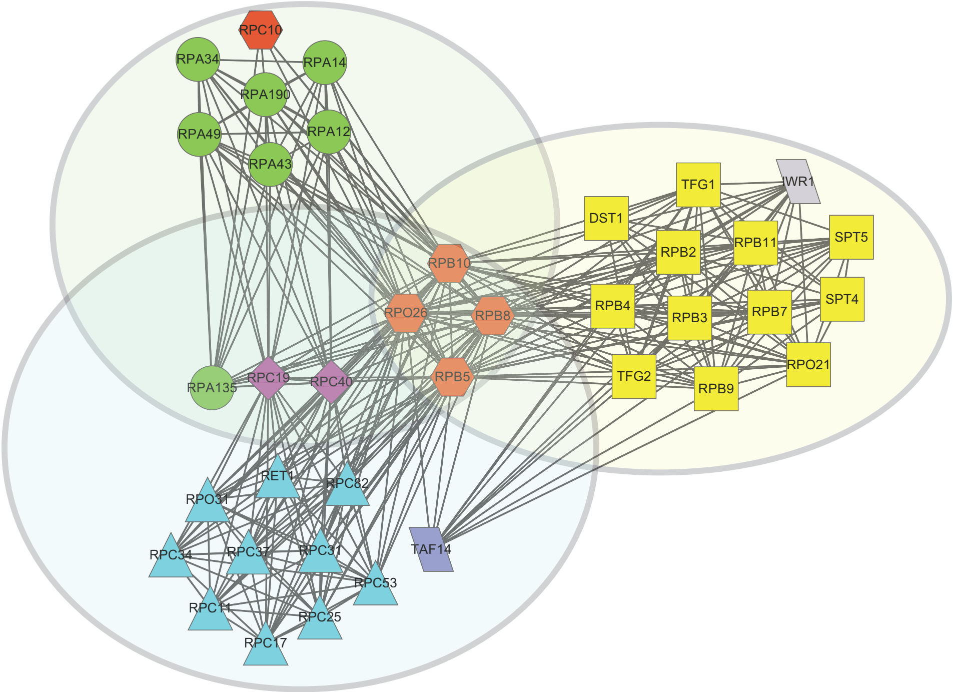

Figure 1: The RNA polymerase I, II, and III detected by CMC on Collins. Proteins are labeled according to the complex they belong to: green circle nodes represent RNA polymerase I, yellow rectangle nodes represent RNA polymerase II, blue triangle nodes represent RNA polymerase III and light purple parallelogram nodes represent proteins with other functions. Proteins shared by all the three complexes are labeled with red hexagon, and proteins shared by RNA polymerase I and III are labeled with purple diamond. Shaded areas represent the clusters detected by CMC. This figure is plotted with software Cytoscape [1].

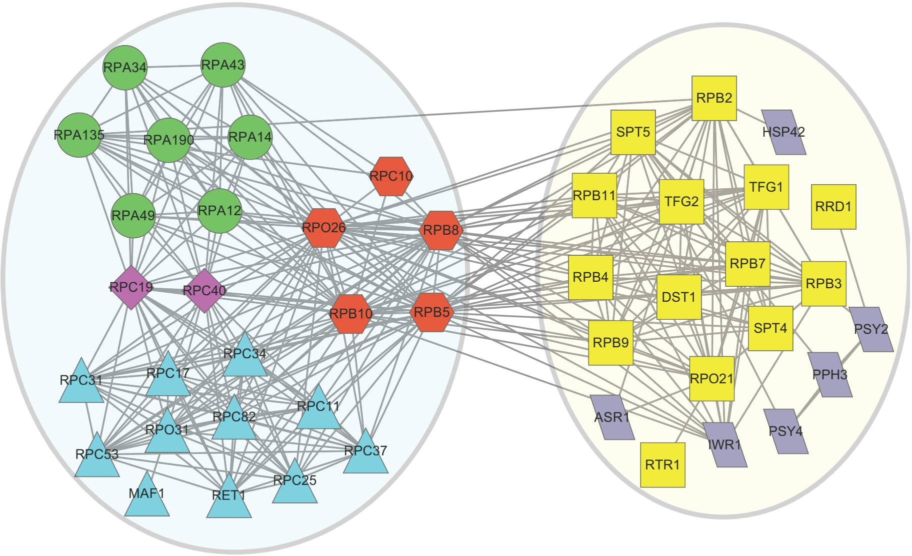

Figure 2: The RNA polymerase I, II, and III detected by COPRA on Collins. Proteins are labeled according to the complex they belong to: green circle nodes represent R-NA polymerase I, yellow rectangle nodes represent RNA polymerase II, blue triangle nodes represent RNA polymerase III and light purple parallelogram nodes represent proteins with other functions. Proteins shared by all the three complexes are labeled with red hexagon, and proteins shared by RNA polymerase I and III are labeled with purple diamond. Shaded areas represent the clusters detected by COPRA. This figure is plotted with software Cytoscape [1].

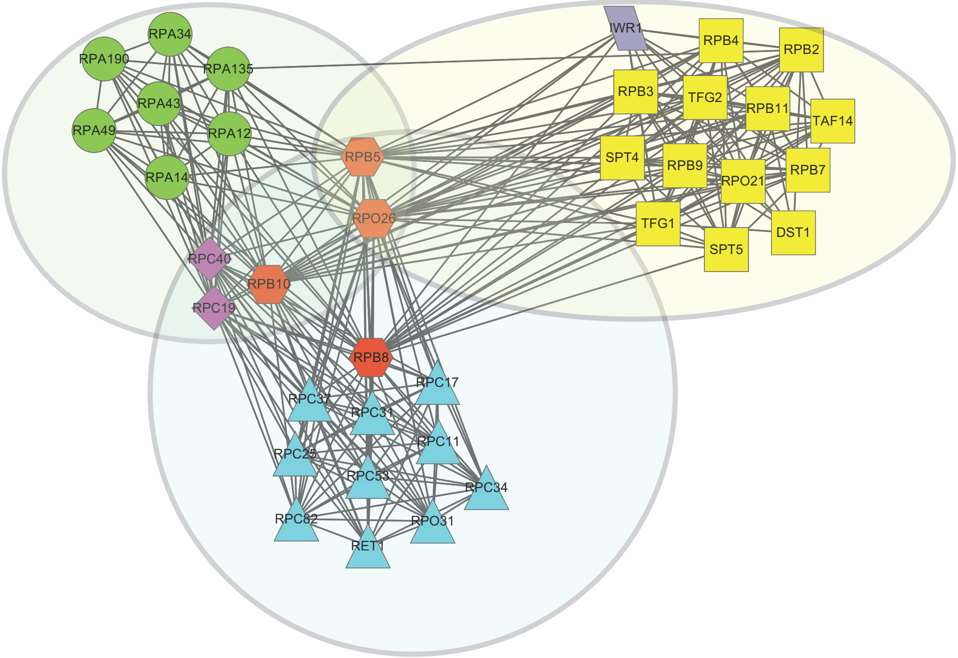

Figure 3: The RNA polymerase I, II, and III detected by DPClus on Collins. Proteins are labeled according to the complex they belong to: green circle nodes represent RNA polymerase I, yellow rectangle nodes represent RNA polymerase II, blue triangle nodes represent RNA polymerase III and light purple parallelogram nodes represent proteins with other functions. Proteins shared by all the three complexes are labeled with red hexagon, and proteins shared by RNA polymerase I and III are labeled with purple diamond. Shaded areas represent the clusters detected by DPClus. This figure is plotted with software Cytoscape [1].

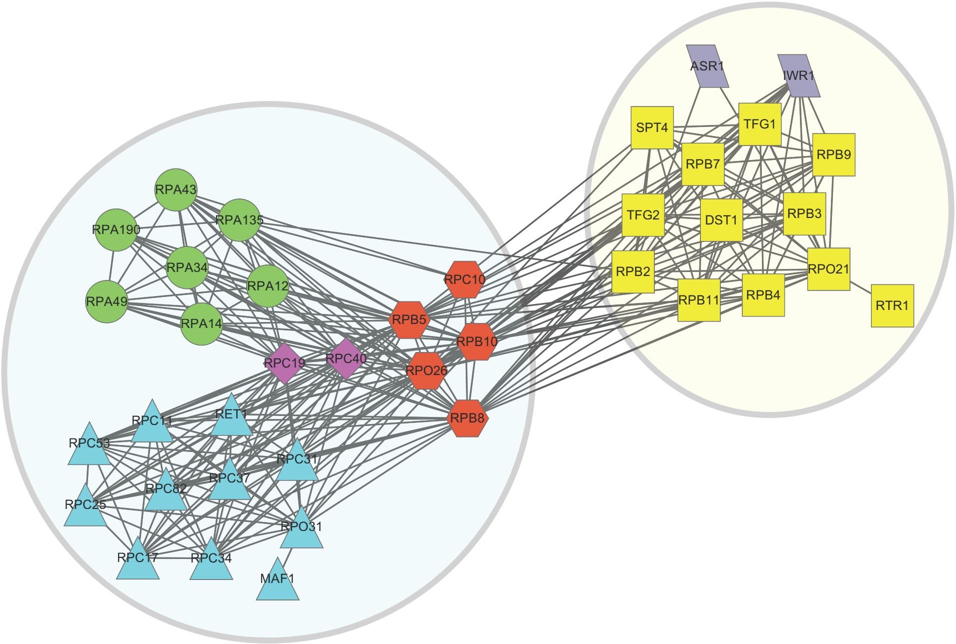

Figure 4: The RNA polymerase I, II, and III detected by MCL on Collins. Proteins are labeled according to the complex they belong to: green circle nodes represent RNA polymerase I, yellow rectangle nodes represent RNA polymerase II, blue triangle nodes represent RNA polymerase III and light purple parallelogram nodes represent proteins with other functions. Proteins shared by all the three complexes are labeled with red hexagon, and proteins shared by RNA polymerase I and III are labeled with purple diamond. Shaded areas represent the clusters detected by MCL. This figure is plotted with software Cytoscape [1].

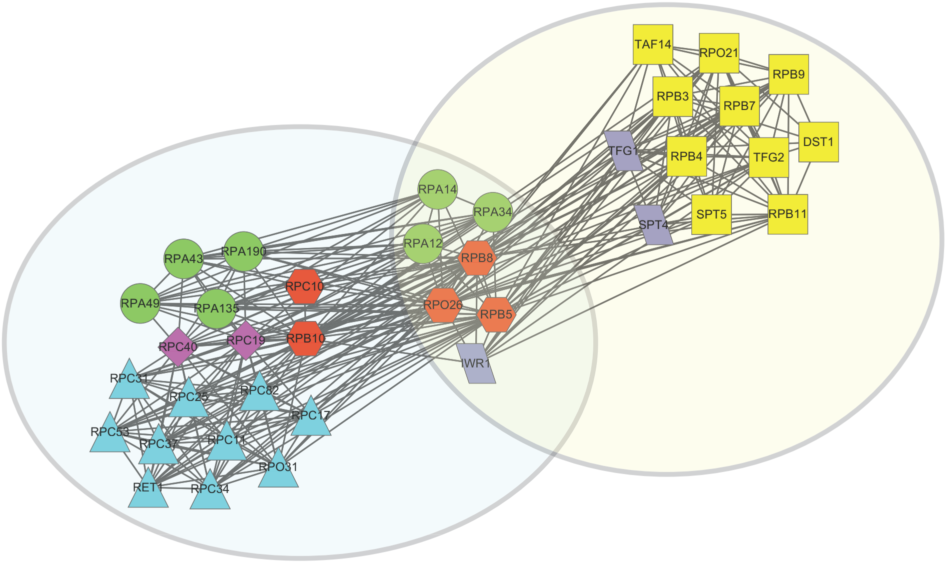

Figure 5: The RNA polymerase I, II, and III detected by MINE on Collins. Proteins are labeled according to the complex they belong to: green circle nodes represent RNA polymerase I, yellow rectangle nodes represent RNA polymerase II, blue triangle nodes represent RNA polymerase III and light purple parallelogram nodes represent proteins with other functions. Proteins shared by all the three complexes are labeled with red hexagon, and proteins shared by RNA polymerase I and III are labeled with purple diamond. Shaded areas represent the clusters detected by MINE. This figure is plotted with software Cytoscape [1].

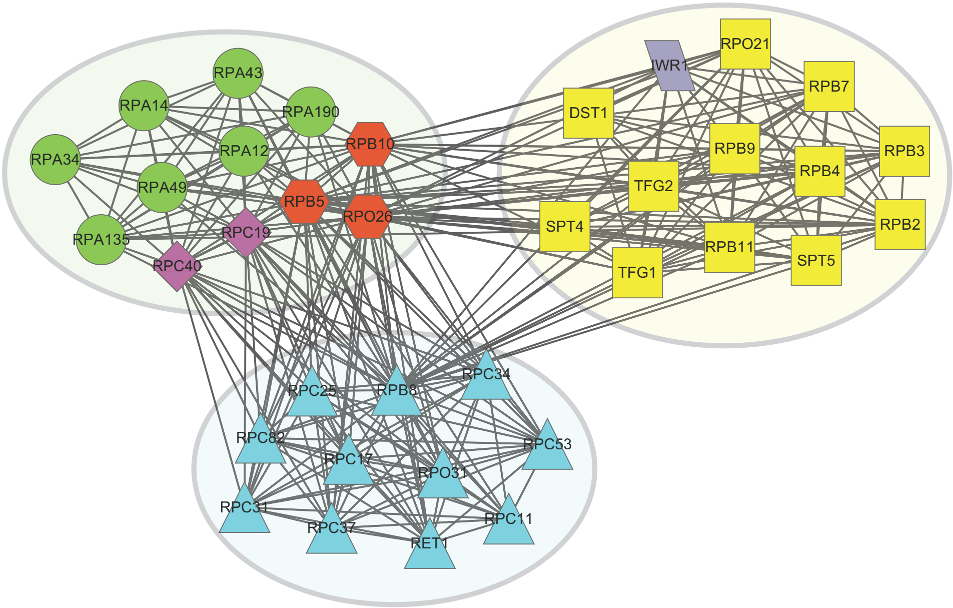

Figure 6: The RNA polymerase I, II, and III detected by RNSC on Collins. Proteins are labeled according to the complex they belong to: green circle nodes represent RNA polymerase I, yellow rectangle nodes represent RNA polymerase II, blue triangle nodes represent RNA polymerase III and light purple parallelogram nodes represent proteins with other functions. Proteins shared by all the three complexes are labeled with red hexagon, and proteins shared by RNA polymerase I and III are labeled with purple diamond. Shaded areas represent the clusters detected by RNSC. This figure is plotted with software Cytoscape [1].

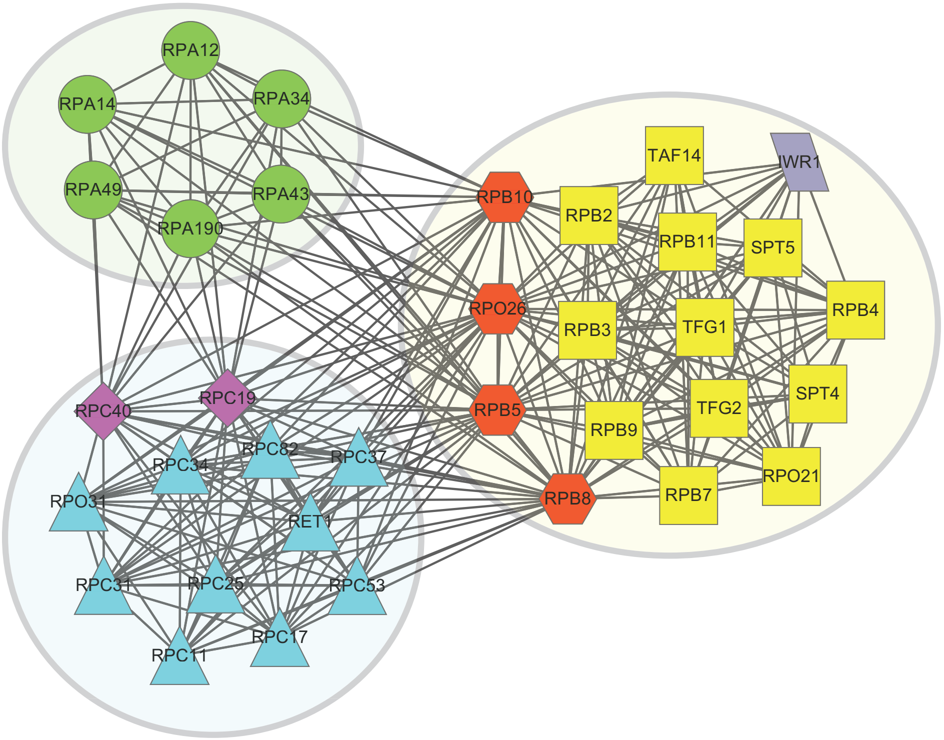

Figure 7: The RNA polymerase I, II, and III detected by SPICi on Collins. Proteins are labeled according to the complex they belong to: green circle nodes represent RNA polymerase I, yellow rectangle nodes represent RNA polymerase II, blue triangle nodes represent RNA polymerase III and light purple parallelogram nodes represent proteins with other functions. Proteins shared by all the three complexes are labeled with red hexagon, and proteins shared by RNA polymerase I and III are labeled with purple diamond. Shaded areas represent the clusters detected by SPICi. This figure is plotted with software Cytoscape [1].

References

[1] M.S. Cline, M. Smoot, E. Cerami, A. Kuchinsky, N. Landys, C. Workman, R. Christmas, I. Avila-Campilo, M. Creech, B. Gross, Kristina Hanspers, Ruth Isserlin, Ryan Kelley, Sarah Killcoyne, Samad Lotia, Steven Maere, John Morris, Keiichiro Ono, Vuk Pavlovic, Alexander R Pico, Aditya Vailaya, Peng-Liang Wang, Annette Adler, Bruce R Conklin, Leroy Hood, Martin Kuiper, Chris Sander, Ilya Schmulevich, Benno Schwikowski, Guy J Warner, Trey Ideker, and Gary D Bader. Integration of biological networks and gene expression data using cytoscape. *Nature Protocols*, 2(10):2366–2382, 2007.
